# Association Between Dietary Total Fat and Cholesterol Intake and Pancreatic Cancer Risk: A Dose–Response Meta-Analysis of Cohort and Case-Control Studies

**DOI:** 10.3390/nu18162674

**Published:** 2026-08-16

**Authors:** Syed O. Ahmad, Musab Umair, Usman Salman Ali, Mohammad Hamza, Emad Kutbi, Saleh A. K. Saleh, Heba M. Adly, Ahmed Abu-Zaid

**Affiliations:** 1 Department of Biochemistry and Molecular Medicine, College of Medicine, Alfaisal University, Riyadh 11533, Saudi Arabia; 2 Department of Internal Medicine, Lowell General Hospital, Lowell, MA 01854, USA; 3 Department of Biorepository, Biomedical Research Administration, King Fahad Medical City, Riyadh 11525, Saudi Arabia; 4 Directorate of Institutional Excellence, Batterjee Medical College, Jeddah 21442, Saudi Arabia; 5 Department of Community Medicine and Pilgrims Healthcare, College of Medicine, Umm Al-Qura University, Makkah 21955, Saudi Arabia

**Keywords:** pancreatic cancer, dietary cholesterol, total fat, observational studies, diet and cancer

## Abstract

**Background:** Pancreatic cancer (PC) is a highly lethal malignancy with a poor prognosis. Dietary total fat and cholesterol intake have been suggested as potential modifiable risk factors, but the evidence remains inconsistent. This study aimed to systematically evaluate the association between dietary total fat and cholesterol intake and PC risk. **Methods:** A comprehensive literature search was conducted in PubMed, Scopus, Google Scholar, and Web of Science from database inception to May 2026. Cohort and case–control studies reporting relative risks (RRs), odds ratios (ORs), or hazard ratios (HRs) for the association between dietary total fat or cholesterol intake and PC risk were included. Pooled effect size (ES) and 95% confidence intervals (CIs) were calculated using random-effects models. **Results:** Twenty-five studies (17 case–control and 8 cohort studies) were included, most of which were of high quality. Cohort studies showed no association of dietary cholesterol (ES: 1.02; 95% CI: 0.87–1.20) or total fat intake (ES: 1.00; 95% CI: 0.84–1.20) with PC risk. In case–control studies, high cholesterol intake was associated with increased PC risk (ES: 1.63; 95% CI: 1.32–2.01), whereas total fat intake was not (ES: 1.18; 95% CI: 0.85–1.65). In cohort studies, dose–response analyses showed no significant associations between cholesterol or total fat intake and PC. In case–control studies, each 100 mg/day increase in cholesterol intake was associated with a 13% higher risk of PC, whereas no significant dose–response association was observed for total fat intake. Subgroup and sensitivity analyses supported these findings, and no publication bias was detected. **Conclusions**: Dietary cholesterol and total fat intake were not associated with PC risk in cohort studies, whereas case–control studies suggested a positive association between cholesterol intake and PC risk. Further prospective studies are needed to clarify these associations.

## 1. Introduction

Pancreatic cancer (PC) is a lethal condition with a five-year survival rate of under 10% [1]. According to GLOBOCAN 2022 statistics, PC ranks 12th in terms of incidence rate and sixth in terms of global mortality among cancers [2]. Approximately 510,566 new cases of PC are diagnosed worldwide annually [2]. Moreover, modelling projections suggest that the global prevalence of PC will continue to rise through 2040, with a steeper increase expected in females and a potentially greater future burden in regions with lower socioeconomic status [3].

The etiology of PC is multifactorial and not fully understood. However, several modifiable and non-modifiable risk factors have been consistently identified, including smoking, heavy alcohol consumption, obesity, chronic pancreatitis, and dietary habits [4,5]. Among dietary factors, the potential roles of total fat and cholesterol intake have attracted substantial research interest over the past decades [6,7,8,9].

Several biologically plausible mechanisms support a link between dietary lipids and PC risk. Experimental evidence from animal models has shown that a high-fat diet in mice with oncogenic Kras expression promotes pancreatic intraepithelial neoplasia formation, fibrosis, inflammation, and progression to pancreatic ductal adenocarcinoma, ultimately reducing survival [10]. Additionally, prolonged high-fat intake may chronically overstimulate pancreatic enzyme secretion, potentially leading to pancreatic hypertrophy or hyperplasia [11]. Dietary cholesterol can affect biliary excretion and cause bile reflux into the head of the pancreas, where most tumors occur [12,13].

Despite these mechanistic insights, epidemiological evidence remains inconsistent. An earlier meta-analysis reported a positive association between dietary cholesterol intake and PC risk globally, except in European populations [13]. In contrast, another meta-analysis found no significant association between total fat intake and PC risk [14]. However, more recent studies have yielded conflicting results: some have shown a positive relationship between total fat intake and PC risk [15,16], while others have not [17,18].

Given these discrepancies and the publication of several new original studies in recent years [6,18,19,20,21], there is a critical need to update and refine the pooled evidence. Although prior meta-analyses evaluated these associations [13], they possessed major methodological limitations; specifically, they combined retrospective case–control and prospective cohort studies into a single pooled estimate without systematic separation in their primary analyses, and they relied solely on comparing the highest versus lowest intake categories without performing dose–response analyses. To address these limitations, we conducted the present systematic review and dose–response meta-analysis.

We hypothesized that higher dietary intake of total fat and cholesterol is associated with an increased risk of PC. The primary objective was to evaluate this association in observational studies. The secondary objective was to quantify the potential dose–response relationship between these dietary exposures and PC incidence. Our findings help to provide clearer evidence for dietary guidelines and public health strategies aimed at reducing PC burden.

## 2. Methods

### 2.1. Study Protocol

The study was carried out in strict adherence to the PRISMA (Preferred Reporting Items for Systematic Reviews and Meta-Analyses) guidelines [22] and the recommendations of the Cochrane Handbook for Systematic Reviews of Interventions.

Prior to commencing the review, the authors developed a predefined study protocol outlining the research question, eligibility criteria, search strategy, study selection process, data extraction procedures, risk of bias assessment, and statistical analysis plan. The protocol was developed internally before the literature search was initiated but was not prospectively registered in PROSPERO.

Ethical approval was not required because the study was based exclusively on data extracted from previously published literature and did not involve the collection of original participant data.

### 2.2. Search Strategy

From database inception to May 2026, we conducted a comprehensive search of PubMed, Scopus, and Web of Science to locate pertinent observational studies. For Google Scholar, the database was searched from inception to May 2026, with only the first 100 citations included. Supplementary Table S1 contains the comprehensive search strategy used in this study. Furthermore, we utilized a hand-search method to manually examine the reference lists of the retrieved articles for any additional studies.

### 2.3. Study Selection and Eligibility Criteria

To identify eligible studies, two independent authors screened titles and abstracts from the systematic search, followed by full-text assessment of potentially relevant articles against predefined eligibility criteria. Discrepancies were resolved by discussion or a third reviewer.

Studies were eligible for inclusion in the meta-analysis if they met the following criteria: (i) observational studies (including cohort, case–control, case–cohort, and nested case–control designs) assessing the association between dietary cholesterol and total fat intake and PC risk; (ii) studies reporting odds ratios (ORs), relative risks (RRs), or hazard ratios (HRs) with corresponding 95% confidence intervals (CIs), or providing sufficient data to calculate these effect estimates; (iii) studies published in English; and (iv) studies involving participants aged 18 years or older.

The following criteria were used for excluding studies from the analysis: (i) animal studies, laboratory studies, review articles, and case reports; (ii) studies on pregnant and lactating women; and (iii) studies with overlapping populations. We used only published studies and did not use gray literature.

### 2.4. Data Extraction

For data extraction, two groups, each consisting of two independent authors, collected the data. Any discrepancies were resolved through communication with the first author. The following details were extracted from each included study: author, year of publication, study design, location, follow-up duration, sample size, age of participants, total fat or cholesterol, exposure assessment method, outcome assessment method, study-specific adjusted estimates of RR, HR, or OR with their 95% CIs, and any matched or adjusted factors in the study design or data analysis.

### 2.5. Quality Assessment

Two reviewers assessed the quality of cohort and case–control studies separately using the Newcastle–Ottawa Scale (NOS) [23]. Discrepancies were resolved through discussions involving a third reviewer to reach a consensus. The NOS assesses the quality of studies based on the following domains: selection of the study groups, comparability of the groups, and ascertainment of the exposure and outcome. Studies were considered high quality if they scored 7 or more stars out of 9 on the NOS.

### 2.6. Statistical Analysis

Study-specific RRs, HRs, and ORs comparing the highest versus lowest categories of dietary intake were extracted as reported in the original studies. Because PC is a relatively rare outcome, these effect measures were considered sufficiently comparable under the rare disease assumption and were pooled directly without mathematical conversion. Accordingly, the summary measure was reported as a pooled effect size (ES). Meta-analyses were performed by pooling the natural logarithms of the reported ES and their corresponding standard errors using random-effects models with the restricted maximum likelihood (REML) estimator [24]. The pooled estimates were then exponentiated and presented as pooled ESs with 95% CI. Heterogeneity was assessed using Cochran’s Q test and the I^2^ statistic. Hence, an I^2^ statistic of less than 25% was considered as indicating low heterogeneity, 25–50% as moderate, between 50 and 75% as high, and above 75% as very high heterogeneity [25]. We conducted a sensitivity analysis to evaluate the impact of individual studies on the overall effect size. This involved removing one study at a time to observe how its exclusion influenced the results of the meta-analysis, ensuring the robustness of our findings. Subgroup analyses were carried out based on various key factors such as location of study, sex, follow-up duration, adjustment for body mass index, and adjustment for energy, in order to identify potential sources of heterogeneity and also report and compare effect sizes across the subgroups. To explore potential sources of heterogeneity, we additionally performed random-effects meta-regression analyses using the restricted maximum likelihood (REML) method with Knapp–Hartung adjustment. Study-level covariates examined in the meta-regression models included follow-up duration, body mass index (BMI) adjustment, and energy intake adjustment.

We employed a method previously outlined [26] to conduct the dose–response analysis. We calculated the slopes (linear trends) and corresponding 95% CI for each individual study by utilizing the natural logarithms of the RR and CI, categorized by total fat and cholesterol. For this method to be applicable, it is necessary to have knowledge of the distribution of cases and person-years (or non-cases) as well as the RRs accompanied by their corresponding variance estimates for a minimum of three quantitative usage categories. We made an approximation of the distribution of cases or person-years in studies that did not provide specific information but instead reported the total number of cases or person-years. This approximation was based on analyzing the data by quantiles, such as dividing the total number of person-years into fifths when examining data by quintiles, to determine the number of person-years in each respective portion. We allocated the median or average level of dietary total fats and cholesterol intake to the RR risk for each study within each category. In instances where studies presented intake ranges, we determined the midpoint of each category by calculating the average of the lower and upper bounds. In cases where the highest category had an open-ended range, we made the assumption that the length of the open-ended interval was equivalent to that of the adjacent interval. On the other hand, if the lowest category had an open-ended range, we assigned the lower boundary of that category as zero. The dose–response results are presented in forest plots, based on a daily increase of 20 g for total fat, and a daily increase of 100 mg for cholesterol. We investigated the possibility of a non-linear dose–response association. To do this, we employed restricted cubic splines with three knots placed at percentiles suggested by Harrell (10%, 50%, and 90%) [27] to model the exposures. The study accounted for the correlation among published risk estimates within each category and utilized a one-stage linear mixed effects meta-analysis to combine the estimates specific to each study [28]. In a single stage, this approach calculates the slope lines specific to each study and merges them to derive a comprehensive average slope [26,29]. It is a superior method compared to the conventional two stage method in terms of precision, flexibility, and efficiency [28].

Publication bias was evaluated using Egger’s [30] and Begg’s tests [31]. Furthermore, funnel plots were visually examined for any signs of asymmetry. If there was any sign of publication bias, we used the trim and fill method to estimate the effect size adjusting for publication bias.

Statistical analyses were conducted using Stata software version 14 (Stata Corp. College Station, TX, USA). A significance level of *p* < 0.05 was used to determine statistical significance for all tests.

## 3. Results

### 3.1. Literature Search and Study Characteristics, and Study Quality

**Figure 1:**
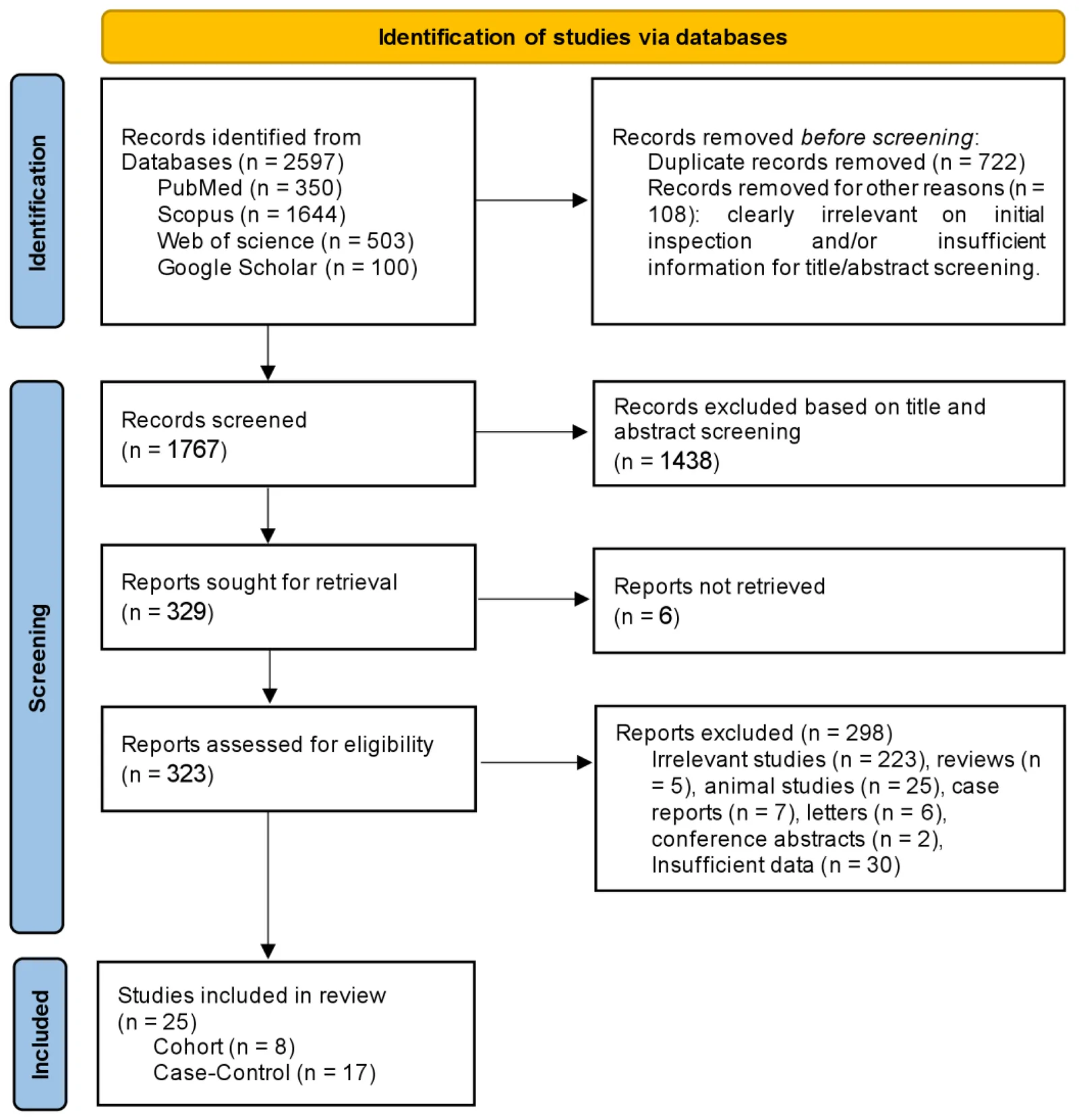
Flow diagram of study selection.

As illustrated in Figure 1, 2597 records were identified from the databases, including PubMed (*n* = 350), Scopus (*n* = 1644), Web of Science (*n* = 503), and Google Scholar (*n* = 100). Following the removal of 722 duplicate records and 108 records removed before screening for other reasons (i.e., clearly irrelevant on initial inspection and/or lacking sufficient information for title/abstract screening), 1767 records were screened by title and abstract. Of these, 1438 records were excluded. Subsequently, 329 reports were sought for retrieval, of which six were not retrieved. The remaining 323 full-text reports were assessed for eligibility, and 298 were excluded based on the predefined criteria, including irrelevant studies (*n* = 223), reviews (*n* = 5), animal studies (*n* = 25), case reports (*n* = 7), letters (*n* = 6), conference abstracts (*n* = 2), and insufficient data (*n* = 30). Finally, 25 studies were included in the review. We identified 17 case–control studies including 3873 cases and 13,746 controls as well as eight prospective studies including 3226 cases and 1,212,706 subjects. Table 1 presents a summary of the characteristics of the studies included in the analysis [6,7,9,12,15,16,17,18,21,32,33,34,35,36,37,38,39,40,41,42,43,44,45,46,47].

The included studies were conducted in North America (*n* = 14) [7,15,17,18,32,33,34,35,36,37,38,39,40,41], Europe (*n* = 7) [9,42,43,44,45,46,47], and others (including Asia and Australia) (*n* = 4) [6,12,16,21]. Age (*n* = 18), smoking (*n* = 23), energy intake (*n* = 20), and history of diabetes (*n* = 14) were adjusted in most studies. Body mass index (*n* = 10) was adjusted in these studies. The follow-up period was longer than 10 years in three cohorts [9,32,42], while it was shorter than 10 years in the remaining five cohorts. Nearly all studies used a food frequency questionnaire (FFQ) to assess dietary exposure.

Study quality was assessed using the NOS. Studies scoring ≥7 out of 9 stars were considered high quality. All eight cohort studies were rated as high quality, with scores ranging from 8 to 9 stars. Among the 17 case–control studies, 12 were classified as high quality, with overall scores ranging from 6 to 9 stars. In total, 20 of the 25 included studies met the criteria for high quality. Detailed item-by-item quality assessments based on the Newcastle–Ottawa Scale for cohort and case–control studies are presented in Supplementary Tables S2 and S3.

### 3.2. Findings on Cholesterol Intake from Cohort Studies

**Figure 2:**
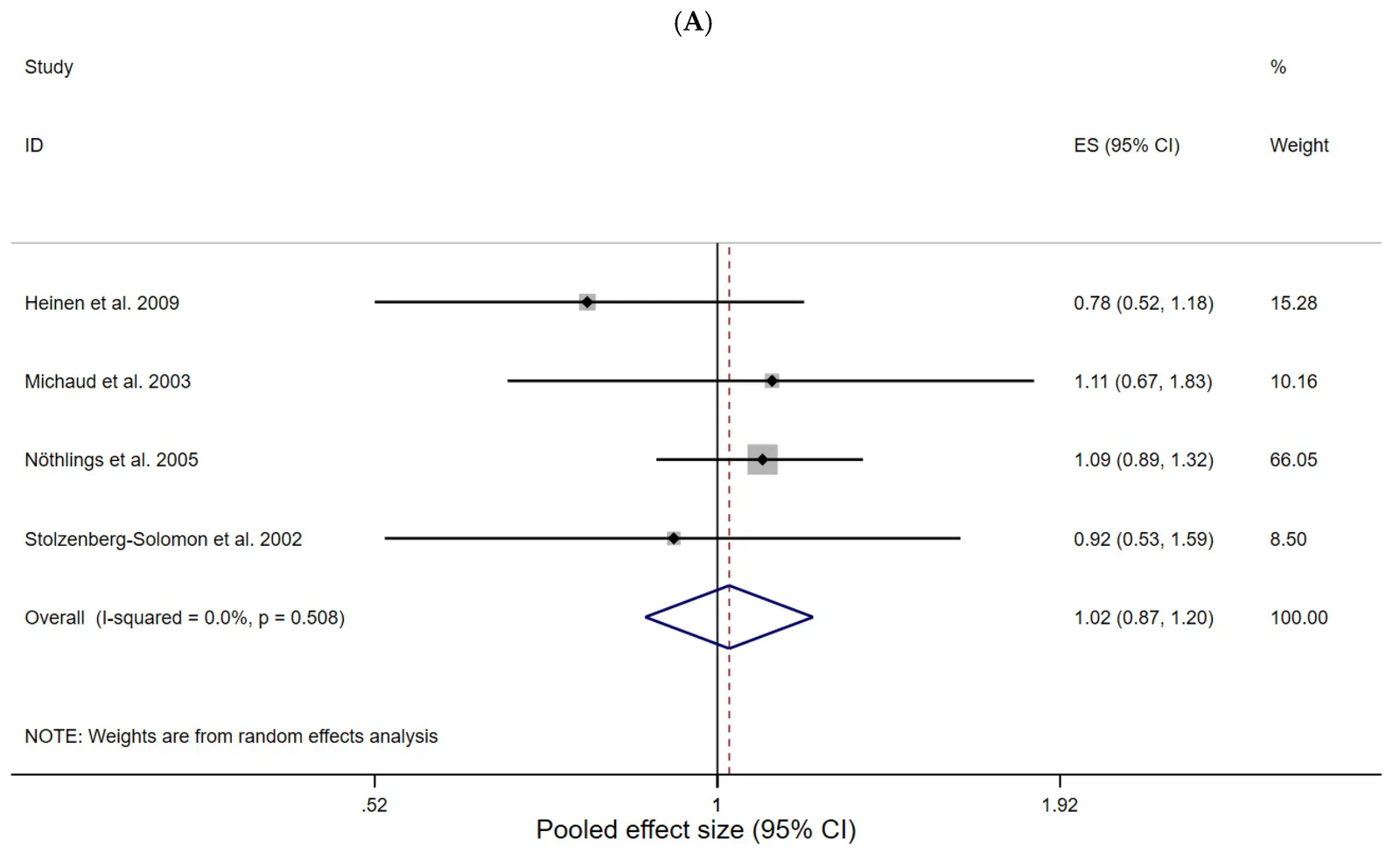

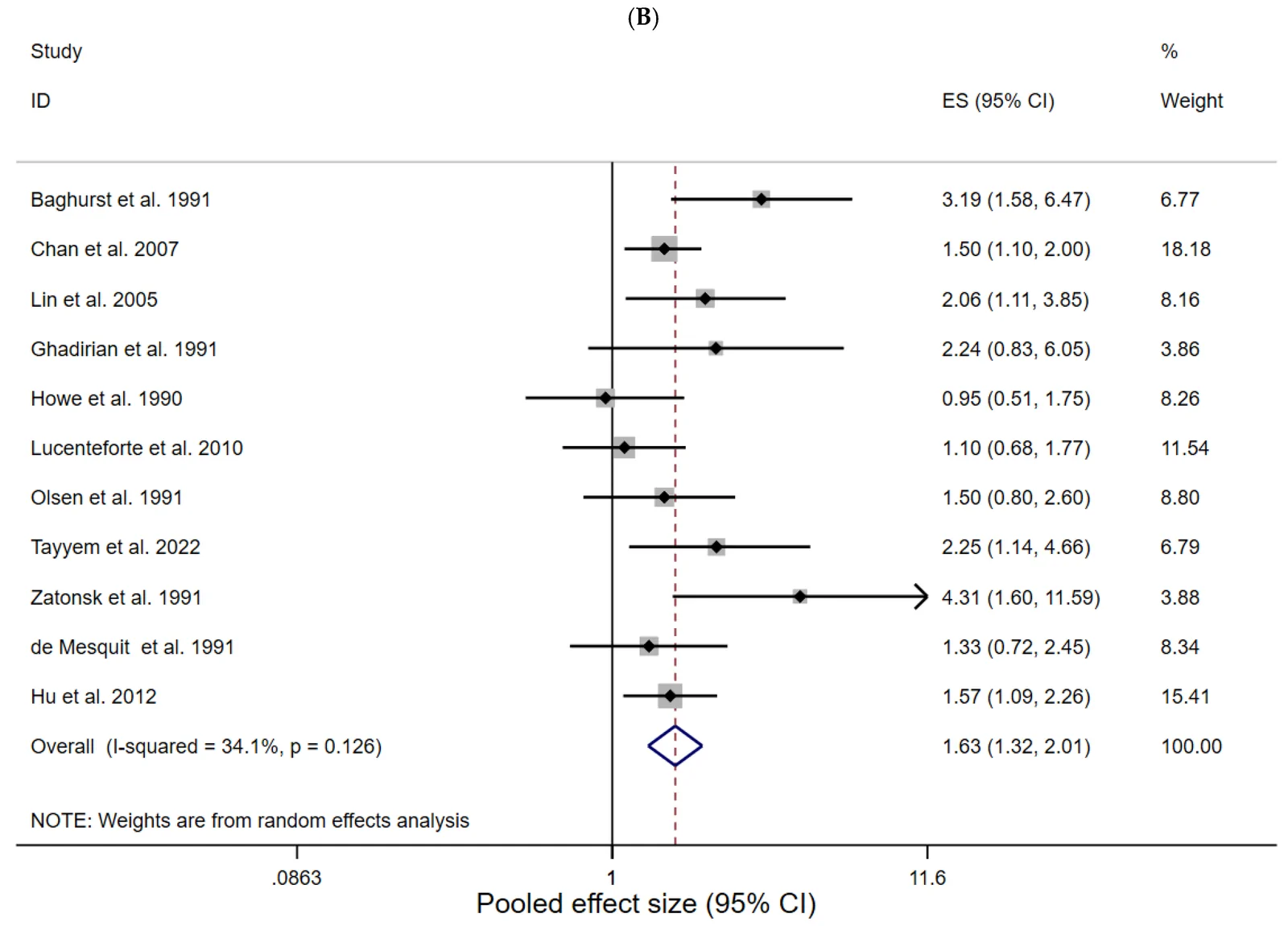
Forest plot showing the association between cholesterol intake (mg/day) and PC risk in adults aged ≥18 years, comparing the highest versus lowest categories of cholesterol intake: cohort studies (**A**) and case–control studies (**B**). The x-axis shows the pooled effect size (ES) with 95% confidence intervals (CIs). Individual study ESs were reported as relative risks (RRs), hazard ratios (HRs), or odds ratios (ORs), as presented in the original studies [9,12,16,21,32,33,35,36,37,38,41,42,44,45,46].

Information on cholesterol intake and PC was available in four cohort studies [9,32,33,42] among 427,147 participants and 1173 cases. There was no significant association between cholesterol intake and PC (ES: 1.02, 95% CI: 0.87–1.20, *p* = 0.78, I^2^ = 0%, P_heterogeneity_ = 0.508) (Figure 2A). There was no evidence of a linear association between cholesterol intake and the risk of PC for each 100 mg/day increase in cholesterol consumption (ES: 1.02, 95% CI: 0.95–1.09, *p* = 0.58, I^2^ = 0%, P_heterogeneity_ = 0.42) (Supplemental Figure S1). No evidence was found to suggest a non-linear association between dietary cholesterol and the incidence of PC (P_non-linearity_ = 0.50, Supplemental Figure S2).

### 3.3. Findings on Cholesterol Intake from Case–Control Studies

The association between cholesterol intake and PC was examined in 11 case–control studies [12,16,21,35,36,37,38,41,44,45,46]. These studies collectively involved 9331 controls and 2549 cases. The results showed that a high intake of dietary cholesterol, compared to the lowest intake, was associated with a 66% higher probability of PC risk in case–control studies (ES: 1.63, 95% CI: 1.32–2.01, *p* < 0.001, I^2^ = 34.1%, P_heterogeneity_ = 0.12) (Figure 2B). The linear dose–response analysis indicated that each 100 mg/day increase in cholesterol intake was associated with a 13% higher risk of PC (ES: 1.13, 95% CI: 1.08–1.18, *p* < 0.001, I^2^ = 4.1%, P_heterogeneity_ = 0.39) (Supplemental Figure S3). The test for non-linearity was not significant (P_non-linearity_ = 0.18, Supplemental Figure S4).

### 3.4. Findings on Total Fat Intake from Cohort Studies

**Figure 3:**
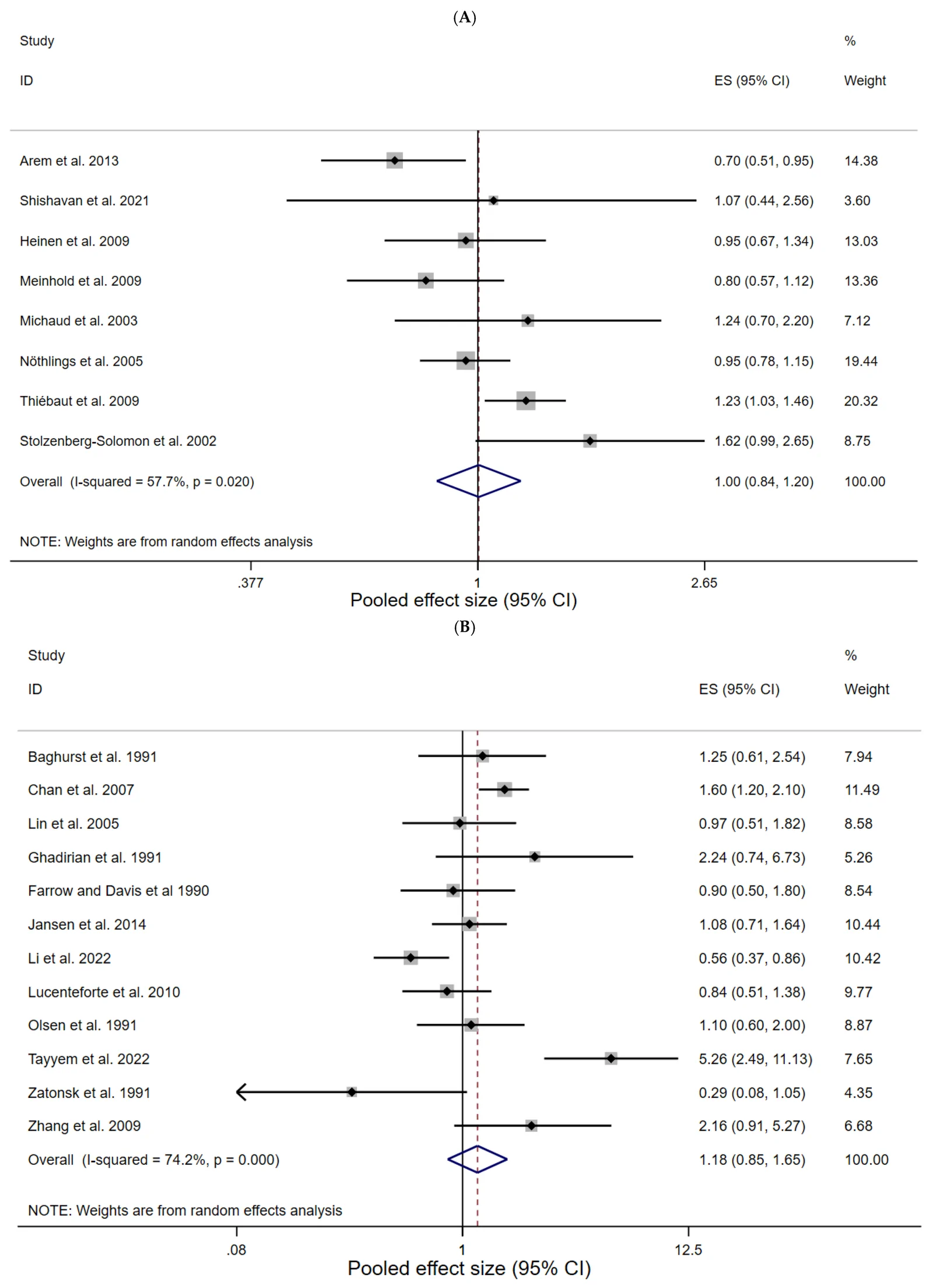
Forest plot for the association between total fat intake (g/day) and risk of PC in adults aged ≥18 years by comparing the highest and lowest categories of total fat intake: cohort studies (**A**), and case–control studies (**B**). Individual study ESs were reported as relative risks (RRs), hazard ratios (HRs), or odds ratios (ORs), as presented in the original studies [6,7,9,12,15,16,17,18,21,32,33,34,35,36,38,39,40,42,44,45].

Eight cohort studies [6,7,9,15,32,33,34,42] with a total of 3263 cases and 1,220,191 participants were included in the analysis of total fat consumption and PC risk. No significant relationship was observed between the highest and lowest daily consumption of total fat and the incidence of PC (ES: 1.00, 95% CI: 0.84–1.20, *p* = 0.96, I^2^ = 57.7%; P_heterogeneity_ = 0.02) (Figure 3A). The linear dose–response analysis showed no significant association between dietary total fat intake and PC risk per 20 g/day increase in total fat consumption (ES: 1.00, 95% CI: 0.95–1.05, *p* = 0.86, I^2^ = 61.5%, P_heterogeneity_ = 0.02) (Supplemental Figure S5). No evidence of a nonlinear association was observed (P_non-linearity_ = 0.93, Supplemental Figure S6).

### 3.5. Findings on Total Fat Intake from Case–Control Studies

Twelve case–control studies [12,16,17,18,21,35,36,38,39,40,44,45] were included in the analysis of total fat intake and PC. These studies included 6450 controls and 3347 cases. The results indicated that there was no significant relationship between the highest versus lowest dietary intake of total fat and the incidence of PC (ES: 1.18, 95% CI: 0.85–1.65, *p* = 0.32, I^2^ = 74.2%; P_heterogeneity_ < 0.0001) (Figure 3B). No linear relationship was found between PC risk and a 20-g/day increase in total fat intake (ES: 1.01, 95% CI: 0.95–1.08, *p* = 0.68, I^2^ = 69.4%, P_heterogeneity_ = 0.02) (Supplemental Figure S7). Also, no evidence was found to indicate a departure from linearity (P_non-linearity_ = 0.27, Supplemental Figure S8).

### 3.6. Subgroup and Sensitivity Analyses

To explore potential sources of between-study differences, we performed subgroup analyses based on sex, study location, duration of follow-up, adjustment for energy intake, and adjustment for body mass index (Table 2). In sensitivity analyses, the summary estimates were not substantially changed.

### 3.7. Publication Bias

**Figure 4:**
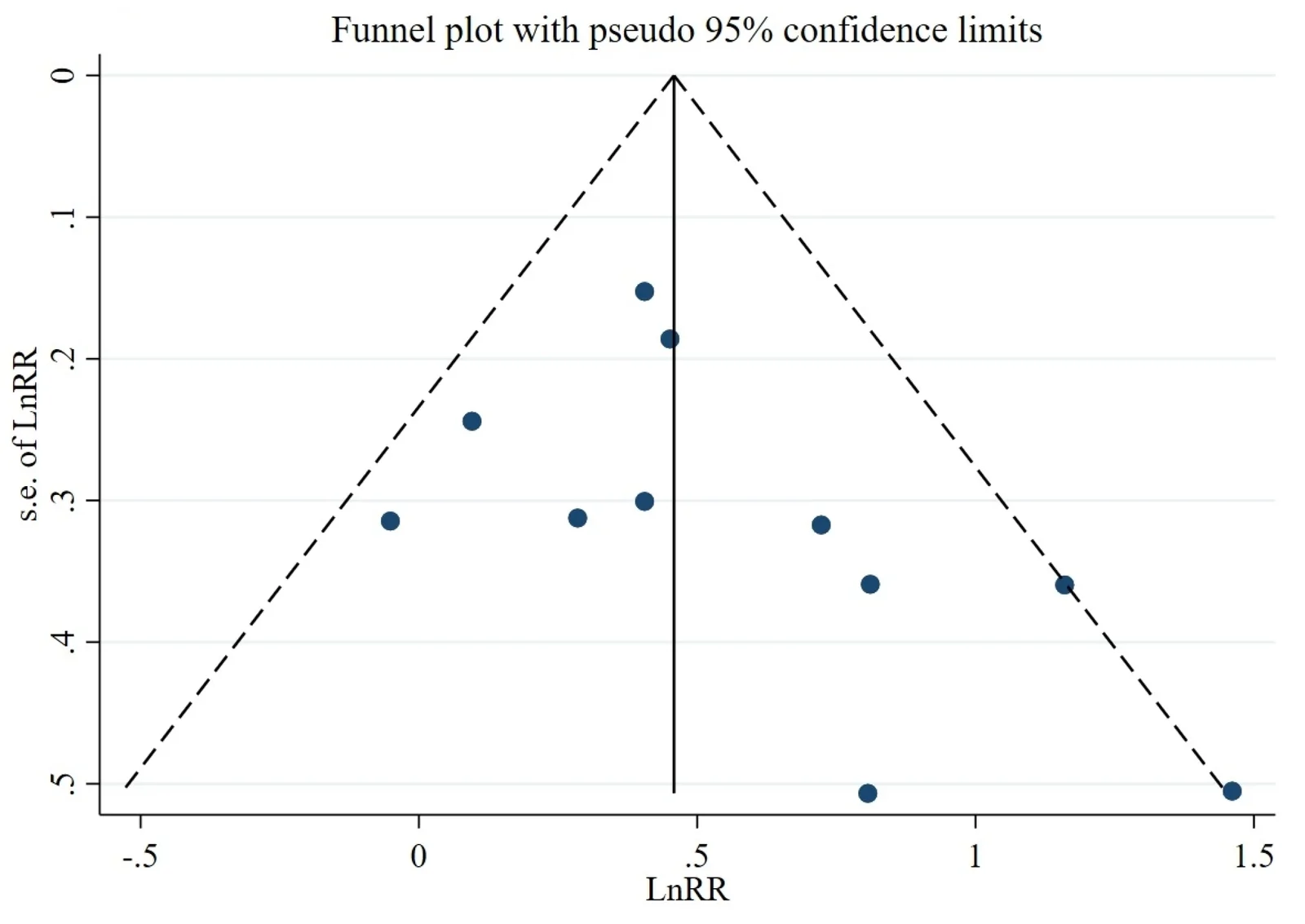
Funnel plot for the association between cholesterol consumption and risk of pancreatic cancer in adults aged ≥18 years in case–control studies. Abbreviations: LnRR, natural logarithm of relative risk; s.e. of LnRR, standard error of the natural logarithm of relative risk.

**Figure 5:**
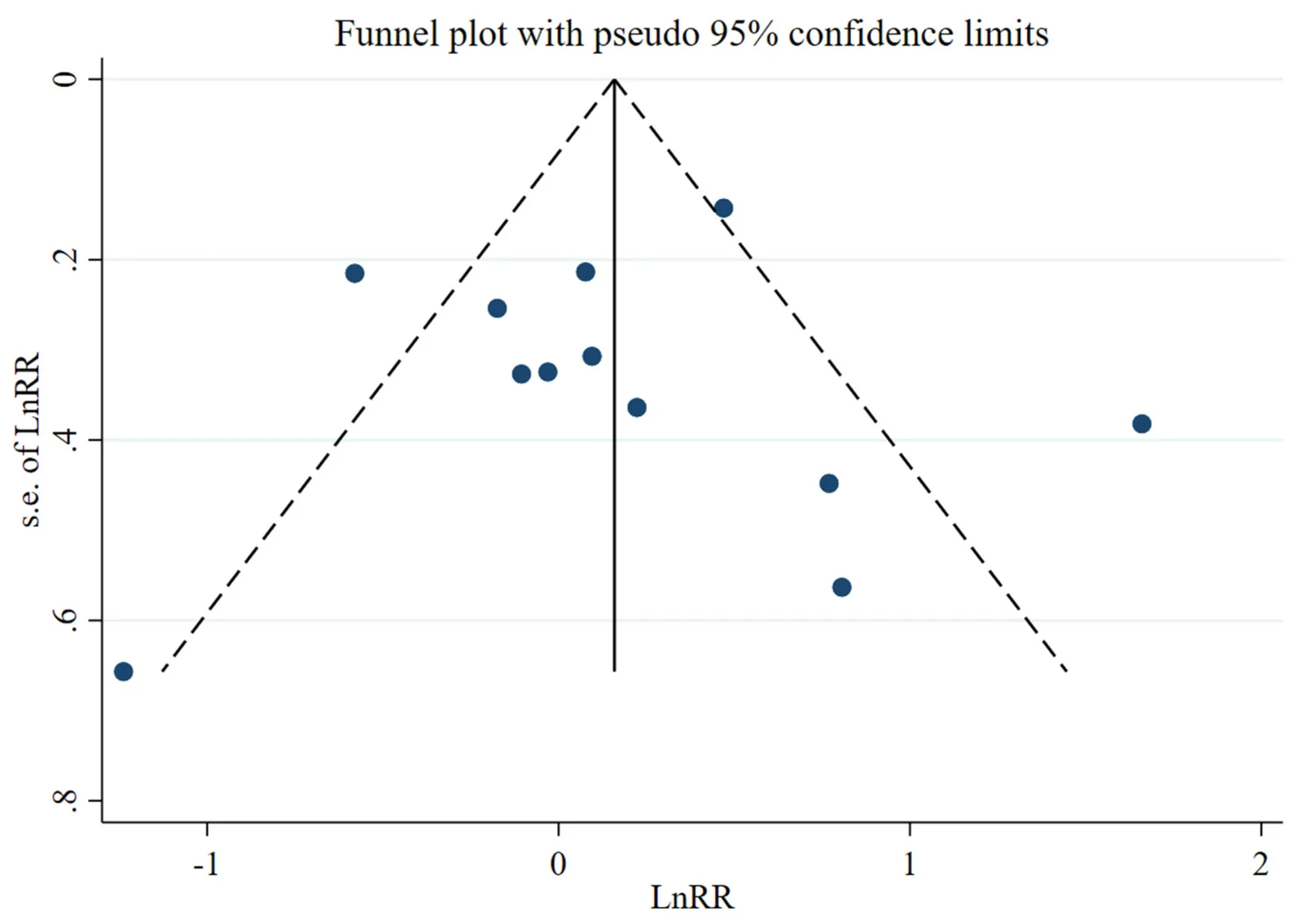
Funnel plot for the association between total fat consumption and risk of pancreatic cancer in adults aged ≥18 years in case–control studies. Abbreviations: LnRR, natural logarithm of relative risk; s.e. of LnRR, standard error of the natural logarithm of relative risk.

There was no evidence of publication bias in the association between cholesterol intake, total fat, and PC in both case–control and cohort studies. We also found no evidence of publication bias for the other associations examined (Figure 4 and Figure 5 for funnel plots).

### 3.8. Meta-Regression

Random-effects meta-regression analyses were conducted to examine whether study-level characteristics explained between-study heterogeneity. In cohort studies of cholesterol intake, neither follow-up duration nor BMI adjustment was significantly associated with the pooled effect estimates (*p* = 0.601, *p* = 0.390, respectively). In case–control studies of cholesterol intake, energy intake adjustment and BMI adjustment were also not significant moderators (*p* = 0.847, *p* = 0.999, respectively). Similarly, in cohort studies of total fat intake, follow-up duration and BMI adjustment were not significantly associated with the effect size (*p* = 0.451, *p* = 0.802, respectively). In case–control studies of total fat intake, BMI adjustment was not a statistically significant moderator of the pooled estimate (*p* = 0.315). Meta-regression plots for these analyses can be found in Supplementary Figures S9 and S12.

## 4. Discussion

### 4.1. Summary of Main Findings

The findings of this meta-analysis, based on 25 observational studies, including eight cohort and 17 case–control studies, showed that higher dietary cholesterol intake was not significantly associated with PC risk in cohort studies. In contrast, a stronger association was observed in case–control studies. These discrepant findings may reflect methodological differences between the two study designs. To the best of our knowledge, this study is the most comprehensive meta-analysis of cohort and case–control studies examining the association between dietary total fat and cholesterol intake and the risk of PC.

### 4.2. Interpretation of Findings and Clinical Implications

An important consideration in interpreting our findings is the clear discrepancy in design between cohort and case–control studies. Prospective cohort studies minimize recall bias because exposure is measured before disease onset, and they are less vulnerable to selection bias. In contrast, retrospective case–control studies rely on participants’ recall of past dietary behaviors, which can be influenced by their disease status, and are prone to selection biases if controls do not represent the source population. Consequently, while case–control studies in our analysis suggested positive associations, these findings must be interpreted with caution. The prospective cohort studies, which provide stronger and higher-level observational evidence, did not show significant associations between dietary total fat or cholesterol intake and PC risk.

Regarding cholesterol intake, the present analysis of case–control studies showed a significant association between the highest and lowest dietary cholesterol intake categories. However, this relationship was not observed in cohort studies, which is consistent with previous findings [13]. Given the inherent susceptibility of retrospective designs to recall and selection biases, these positive findings likely reflect systematic biases rather than true causal relationships.

Several biological mechanisms have been hypothesized to explain a potential link between cholesterol and PC risk, although they remain speculative and are largely based on non-human or in vitro models. As hypothesized, high-fat diets may increase bile acid secretion, and bile acid reflux into the pancreatic duct could induce chronic pancreatitis—a known risk factor for pancreatic carcinogenesis [48]. However, evidence for this mechanism in humans remains inconclusive. Furthermore, while experimental studies suggest that cholesterol metabolites might affect signaling pathways involved in cell proliferation and apoptosis, potentially acting as tumor promoters [49], these effects appear highly context-dependent, and epidemiologic evidence in humans is insufficient to establish a causal pathway.

In the linear dose–response analysis of case–control studies, each 100 mg/day increase in total cholesterol intake was associated with a 13% increase in PC incidence. Nevertheless, this finding should be interpreted cautiously because observational studies, especially case–control studies, are susceptible to selection and recall biases, which may inflate the apparent association between diet and disease [50]. Future well-designed prospective cohort studies are needed to clarify the exact relationship between cholesterol consumption and PC risk.

Our findings were also consistent with a previous study that reported a non-significant relationship between total fat intake and PC risk [14]. It must be kept in mind that total fat consists of several fatty acids, including PUFA, MUFA, SFA, and TFA, which affect the risk of PC differently [51]. There is growing evidence emphasizing the quality of fatty acids rather than their quantity [52]. Therefore, in examining the relationship between diseases and dietary fat intake, not only should total fat be considered but also the types of fatty acids should be addressed separately.

The subgroup analysis stratified by BMI adjustment found no association between PC and total fat intake. However, obesity, a common consequence of high-fat diets, may potentially mediate the relationship with PC risk by influencing chronic inflammation, increasing insulin resistance, and altering adipokines. These factors might be involved in PC development through mechanisms related to insulin-like growth factor (IGF) signaling and inflammatory pathways. Increased insulin levels have been suggested to potentially influence PC development through IGF signaling [53,54].

Although case–control studies suggested positive associations, these findings should be interpreted cautiously because retrospective dietary assessment is more vulnerable to recall bias, reverse causation, and exposure misclassification. In contrast, the prospective cohort studies, which provide stronger evidence, did not show significant associations between dietary total fat or cholesterol intake and PC risk.

Several methodological differences across the primary studies may explain the substantial discrepancies in findings between study designs. First, differences in dietary assessment methods, such as the use of non-validated food frequency questionnaires (FFQs) or diet histories, introduce measurement errors. In retrospective case–control studies, this is compounded by recall bias, where cases may overreport fat or cholesterol intake. Second, exposure categorization differed significantly, as studies compared different quantile thresholds (tertiles, quartiles, or quintiles), making risk estimate comparisons challenging. Third, adjustment for confounding factors was inconsistent; while some studies adjusted for key confounders like total energy intake and BMI, others did not, leaving room for residual confounding. Finally, the studies spanned several decades (from 1990 onwards), during which general dietary habits, food preparation methods, and the fat content of commercial foods changed. Because most studies relied on a single baseline dietary assessment, they could not account for these secular trends or individual longitudinal changes in dietary habits, which may have attenuated any potential associations in cohort studies.

From a public health and clinical perspective, the practical significance of our findings is limited. Since the most robust observational evidence from prospective cohort studies does not support an association between dietary total fat or cholesterol intake and PC risk, these dietary components cannot be considered established risk factors for pancreatic cancer. Consequently, the current body of evidence does not justify recommending reductions in dietary cholesterol or total fat intake specifically for the prevention of PC. While general dietary guidelines continue to recommend moderate fat consumption for cardiovascular and metabolic health, targeted dietary modifications for PC prevention are not supported.

### 4.3. Study Strengths and Limitations

The present study has several notable strengths. It included a large sample size combining prospective cohort and case–control studies, with adjustment for numerous potential confounding variables. No evidence of publication bias was detected, and both linear and dose–response analyses were performed, providing more comprehensive and informative results.

Nonetheless, this study has several limitations. First, most included studies assessed dietary intake using FFQs, which are prone to measurement error due to reliance on participant memory and long recall periods. Second, the lack of information on average fat intake levels across categories, different units used for reporting fat consumption across studies, and measuring fat intake only at baseline in some studies limited our dose–response analyses. Additionally, our meta-analysis is limited by the inherent methodological heterogeneity of the primary literature. This includes variations in dietary assessment tools, exposure categorizations, and confounding adjustment strategies, as well as the inability to account for longitudinal shifts in dietary habits over the extended study periods. Third, case–control studies—which comprised the majority of included studies—are inherently subject to recall and selection biases, and their findings should be interpreted as preliminary evidence rather than causal relationships [55]. In contrast, prospective cohort studies provide higher-level evidence but had smaller sample sizes in our analysis, which may contribute to variability in pooled estimates. Fourth, the generalizability of our findings is limited, as no studies were available from underrepresented regions such as Africa and South America. A further limitation of this study is that, although a predefined review protocol was developed before the review was conducted, it was not prospectively registered in PROSPERO, which may have reduced the transparency of the review process. Finally, restricting inclusion to English-language studies may have introduced language bias and may have resulted in the omission of relevant non-English reports.

### 4.4. Future Research Directions

To address these limitations and better understand the potential biological associations, we recommend several approaches. Prospective cohort studies with long-term follow-up and repeated, detailed dietary assessments are needed to accurately track dietary changes over time. Mendelian randomization studies using genetic variants linked to fat metabolism represent a promising approach to minimize confounding and reverse causation. Well-designed case–control studies with representative controls and comprehensive lifetime dietary data would also improve accuracy. Additionally, future research should include diverse populations from underrepresented regions and prioritize standardized dietary assessment and reporting methods. Incorporating a comprehensive dietary quality index may offer insights into combined effects of dietary patterns on PC risk, complementing single-nutrient approaches.

## 5. Conclusions

This meta-analysis found no significant association between dietary total fat intake or cholesterol intake and PC risk in prospective cohort studies, which provide the highest level of observational evidence. Although case–control studies suggested a positive association between high cholesterol intake and PC risk, these findings should be interpreted with caution as they are likely influenced by recall and selection biases inherent to retrospective study designs. Therefore, the current body of evidence does not provide strong support for dietary cholesterol or total fat as independent risk factors for PC. Well-designed prospective studies with repeated dietary assessments, standardized reporting, and diverse populations are needed to clarify these associations.

## Figures and Tables

**Table 1:** Characteristics of included studies on the association between dietary fat consumption and pancreas cancer in adults aged >18 years.

Study ID	Design	Country	Age	*n*	Follow Up	Cases	Exposure	Exposure Assessment	Comparison	Effect Size (95% CI)	Adjustment	NOS
Heinen et al. 2009 [9]	Cohort	Netherlands	55–69	M/F: 120,852	13.3	350	Cholesterol (mg/d)	FFQ	High vs. low Per 100 mg/d	RR: 0.78 (0.52–1.18) RR: 0.93 (0.80–1.09)	Age, sex, BMI, energy, smoking, alcohol, history of DM, history of hypertension, vegetables, and fruits intake	9
							Total Fat (g/d)		High vs. low 10 g/d intake increment	RR: 0.95 (0.67–1.34) RR: 0.97 (0.88–1.06)		
Michaud et al. 2003 [32]	Cohort	USA	30–55	F: 88,802	18	178	Cholesterol (mg/d)	FFQ	>466 vs. <212	HR: 1.11 (0.67–1.83)	Pack-years of smoking, BMI, history of DM, caloric intake, height, physical activity, menopausal status, and glycemic load intake	9
							Total Fat (g/d)		>87 vs. <52	HR: 1.24 (0.70–2.20)		
Nöthlings et al. 2005 [33]	Cohort	USA	45–75	F/M: 190,545	7	482	Cholesterol (mg/1000 kcal per day)	Quantitative FFQ	>156.8 vs. <56.8	HR: 1.09 (0.89–1.32)	Age, ethnicity, history of DM, familial history of pancreatic cancer, smoking status and energy intake	8
							Total Fat (g/d)		>43.3 vs. <22.7	HR: 0.95 (0.78–1.15)		
Stolzenberg Solomon et al. 2002 [42]	Cohort	Finland	50–69	F/M: 26,948	13	163	Cholesterol (mg/d)	DHQ	High vs. low	HR: 0.92 (0.53–1.59)	Energy intake, age, years of smoking and energy- adjusted saturated fat intake	9
							Total Fat (g/d)			HR: 1.62 (0.99–2.65)		
Shishavan et al. 2021 [6]	Cohort	Iran	40–75	F/M: 46,980	4	76	Total Fat (g/d)	FFQ	>88.2 vs. <60.03 per 16.79 g/d	HR: 1.07 (0.44–2.59) HR: 1.00 (0.98–1.02)	Energy and total fat intake, BMI, age, sex, marital status, residence, smoking, opium consumption, diabetes, physical activity, family history of cancer, ethnicity, wealth score and education	8
Arem et al. 2013 [7]	Cohort	USA	55–74	F/M: 111,416	8.4	411	Total Fat (g/d)	DHQ	>46.9 vs. <23.4	HR: 0.70 (0.51–0.95)	Age, gender, calories, BMI, smoking status, and DM	9
Thiébaut et al. 2009 [15]	Cohort	USA	50–71	F/M: 525,473	6.3	1337	Total Fat (g/d)	DHQ/FFQ	High vs. low	HR: 1.23 (1.03–1.46)	Age, sex, total energy intake, BMI, smoking history, and DM	8
Meinhold et al. 2009 [34]	Cohort	USA	55–74	M/F: 101,690	6.5	229	Total Fat (g/d)	FFQ	>45.6 vs. <24.3	HR: 0.80 (0.57–1.12)	Age, BMI, number of cigarettes/day, years of smoking, total calories, and DM	8
Baghurst et al. 1991 [12]	Case–control	Australia	50–80	M/F: 357	NA	104	Cholesterol (mg/d)	FFQ	High vs. low	RR: 3.19 (1.57–6.47)	Total energy intake, alcohol consumption, and tobacco usage.	7
							Total Fat (g/d)			RR: 1.25 (0.61–2.54)		
Chan et al. 2007 [35]	Case–control	USA	50–80	M/F: 2233	NA	532	Cholesterol (mg/d)	Semi-quantitative FFQ	>368.9 vs. <122.8	OR: 1.50 (1.10–2.00)	Education, smoking, BMI, diabetes, and energy intake	9
							Total Fat (g/d)		>89.7 vs. <32.1	OR: 1.60 (1.20–2.10)		
Lin et al. 2005 [16]	Case–control	Japan	40–79	M/F: 327	NA	109	Cholesterol (mg/d)	FFQ	>330 vs. <206	OR: 2.06 (1.11–3.85)	Energy intake by the residual method	7
							Total fat (g/d)		>58.6 vs. <44.8	OR: 0.97 (0.51–1.82)		
Ghadirian et al. 1991 [36]	Case–control	Canada	35–79	M/F: 418	NA	179	Cholesterol (mg/d)	FFQ	High vs. low	OR: 2.24 (0.83–6.05)	Age, sex, place of residence, Lifetime cigarette smoking, response status	6
							Total Fat (g/d)			OR: 2.97 (1.02–8.62)		
Howe et al. 1990 [37]	Case–control	Canada	35–79	M/F: 754	NA	249	Cholesterol (mg/d)	Quantitative dietary history	High vs. low	RR: 0.95 (0.51–1.75)	Caloric and fiber intake, lifetime cigarette consumption	8
Kalapothaki et al. 1993 [43]	Case–control	Greece	NA	M/F: 362	NA	181	Cholesterol (mg/d)	FFQ	Per 70 mg/d	RR: 1.19 (0.96–1.47)	Age, Gender, Hospital past residence, years of schooling, cigarette smoking, DM	7
							Total Fat (g/d)		Per 10 g/d	RR: 1.03 (0.87–1.22)		
Lucenteforte et al. 2010 [44]	Case–control	Italy	34–80	M/F: 978	NA	326	Cholesterol (mg/d)	FFQ	High vs. low	OR: 1.10 (0.68–1.77)	Year of interview, education, tobacco smoking, history of diabetes, and total energy intake, according to the residual mode	8
							Total Fat (g/d)		>108.5 vs. <55.5 Per 13.25 g/d	OR: 0.84 (0.51–1.38) OR: 0.92 (0.79–1.08)		
Olsen et al. 1991 [38]	Case–control	USA	40–84	M: 432	NA	212	Cholesterol (mg/d)	FFQ	High vs. low	OR: 1.50 (0.80–2.60)	Total energy intake, age, cigarette usage, alcohol consumption, respondent-reported history of DM, and educational level	7
							Total Fat (g/d)			OR: 1.10 (0.60–2.00)		
Tayyem et al. 2022 [21]	Case–control	Jordan	NA	M/F: 409	NA	100	Cholesterol (mg/d)	FFQ	High vs. low	OR: 2.25 (1.14–4.66)	Total energy intake, age, sex, marital status, education, BMI before the diagnosis of PC, smoking, personal history of diabetes, and physical activity.	8
							Total Fat (g/d)			OR: 5.26 (2.49–11.13)		
Zatonsk et al. 1991 [45]	Case–control	Poland	NA	M/F: 305	NA	110	Cholesterol (mg/d)	FFQ	High vs. low	RR: 4.31 (1.6–11.59)	Cigarette lifetime consumption and calories	6
							Total Fat (g/d)			RR: 0.29 (0.08–1.05)		
de Mesquit et al. 1991 [46]	Case–control	Netherlands	NA	M/F: 644	NA	164	Cholesterol (mg/d)	FFQ	High vs. low	OR: 1.33 (0.72–2.45)	Age, sex, response status, total smoking, and dietary intake of energy	6
Jansen et al. 2014 [17]	Case–control	USA	NA	M/F: 1367	NA	384	Total Fat (g/d)	FFQ	>39.27 vs. <27.9	OR: 1.08 (0.71–1.64)	Age, sex, cigarette smoking, BMI, DM, energy intake, alcohol, and daily servings of total fruit and vegetable consumption.	7
Bueno de Mesquit et al. 1990(A) [47]	Case–control	Netherlands	35–79	M: 412	NA	90	Total Fat (g/d)	FFQ	Per 72 g/d	OR: 0.15 (0.03–0.75)	Age, sex, response status, total smoking	6
Bueno de Mesquit et al. 1990(B) [47]	Case–control	Netherlands	35–79	F: 322	NA	74	Total Fat (g/d)	FFQ	Per 56 g/d	OR: 1.36 (0.31–6.02)	Age, sex, response status, total smoking	6
Li et al. 2022 [18]	Case–control	USA	50–70	M/F: 1558	NA	957	Total Fat (g/d)	FFQ	High vs. low	OR: 0.56 (0.37–0.86)	Energy, education, smoking, alcohol, diabetes, family history of cancer, carbohydrate and protein intakes	9
Farrow and Davis et al. 1990 [39]	Case–control	USA	30–74	M/F: 336	NA	148	Total Fat (g/d)	DHQ	High vs. low	OR: 0.90 (0.50–1.80)	Age, smoking, education, and calorie-adjusted calcium intake	8
Hu et al. 2012 [41]	Case–control	Canada	20–76	M/F: 5667	NA	628	Cholesterol (mg/d)	FFQ	High vs. low	OR: 1.57 (1.09–2.26)	Sex, Age group, province, education, BMI, alcohol drinking (grams/day), pack year smoking, total vegetable and fruit intake (servings/week), saturated fat (servings/week) and total energy intake	8
Zhang et al. 2009 [40]	Case–control	USA	20–64	M/F: 738	NA	186	Total Fat (g/d)	FFQ	High vs. low	OR: 2.16 (0.91–5.27)	Age, sex, race, education, cigarette smoking, alcohol intake, physical activity, and intakes of all other dietary factors; saturated fat, monounsaturated fat, polyunsaturated fat, and total fat	8

Abbreviations: BMI: body mass index; RR: relative risk; FFQ: food frequency questionnaire; M: male; F: female; g/d: gram(s) per day; HR: hazard ratio; OR: odds ratio; DM: diabetes mellitus; NA: not applicable; NOS: Newcastle–Ottawa Scale.

**Table 2:** Stratified analyses on associations of dietary fats consumption with risk of pancreatic cancer (PC) in adults aged >18 years.

		*n*	Pooled ES (95% CI)	I^2^ (%)	P-Heterogeneity
Cohort studies Subgroup analyses of cholesterol					
Location					
	US	2	1.09 (0.91–1.31)	0	0.94
	Non-US	2	0.83 (0.60–1.15)	0	0.63
Sex					
	Male and female	3	1.00 (0.83–1.21)	9.6	0.33
	Male	1	1.11 (0.67–1.83)	0	-
	Female	0	-	-	-
Follow-up duration					
	>10 years	3	0.90 (0.69–1.19)	0	0.56
	<10 years	1	1.09 (0.90–1.33)	0	-
Adjustment for energy					
	Yes	4	1.02 (0.87–1.20)	0	0.50
	No	0	-	-	-
Adjustment for BMI					
	Yes	4	1.02 (0.87–1.20)	0	0.50
	No	0	-	0	-
Subgroup analyses of total fat					
Location					
	US	5	0.95 (0.76–1.19)	69.1	0.01
	Non-US	3	1.16 (0.81–1.67)	34.1	0.21
Sex					
	Male and female	7	0.99 (0.81–1.20)	62.8	0.01
	Male	0	-	-	-
	Female	1	1.24 (0.70–2.20)	0	-
Follow-up duration					
	>10 years	3	1.19 (0.86–1.65)	35.4	0.21
	<10 years	5	0.93 (0.74–1.17)	67.8	0.01
Adjustment for energy					
	Yes	7	1.00 (0.83–1.21)	63.8	0.01
	No	1	1.07 (0.44–2.56)	-	-
Adjustment for BMI					
	Yes	8	1.00 (0.84–1.20)	57.7	0.02
	No	–	-	-	-
Case–control studies Subgroup analyses of cholesterol					
Location					
	US	2	1.50 (1.15–1.96)	0	1.00
	Non-US	9	1.72 (1.29–2.29)	46.5	0.06
Sex					
	Male and female	10	1.66 (1.31–2.09)	40.6	0.08
	Male	1	1.50 (0.83–2.70)	0	-
	Female	0	-	-	-
Adjustment for energy					
	Yes	10	1.67 (1.33–2.10)	46.5	0.06
	No	1	1.33 (0.72–2.45)	-	-
Adjustment for BMI					
	Yes	6	1.55 (1.29–1.87)	62.4	0.03
	No	5	1.78 (1.09–2.93)	0	0.48
Subgroup analyses of total fat					
Location					
	US	6	1.09 (0.74–1.61)	73.9	0.002
	Non-US	6	1.28 (0.66–2.51)	78.5	<0.001
Sex					
	Male and female	11	1.19 (0.83–1.71)	76.5	<0.001
	Male	1	1.10 (0.60–2.01)	-	-
	Female	0	-	-	-
Adjustment for energy					
	Yes	12	1.18 (0.85–1.65)	74.2	<0.001
	No	0	-	-	-
Adjustment for BMI					
	Yes	5	1.51 (0.81–2.82)	87.7	<0.001
	No	7	1.00 (0.75–1.34)	20.6	0.27

Abbreviations: CI, confidence interval; ES: effect size.

## Data Availability

All data are available within the manuscript and its Supplemental Files.

## Supplementary Materials

The following supporting information can be downloaded at: https://www.mdpi.com/article/10.3390/nu18162674/s1, Table S1: Search strategy for searching articles in scientific databases; Table S2: The quality of cohort studies using the Newcastle-Ottawa Scale; Table S3: The quality of case-control studies using the Newcastle-Ottawa Scale; Figure S1: Forest plot for the risk of pancreatic cancer based on 100 mg/day increase in cholesterol intake in adults aged >18 years. Horizontal lines represent 95% CIs. Diamonds represent the pooled estimates from the random-effects analysis. ES: effect size, CI: confidence interval; Figure S2: Non-linear analysis of cholesterol intake with risk of pancreatic cancer in adults over 18 years of age among cohort studies; Figure S3: Forest plot for the risk of pancreatic cancer based on 100 mg/day increase in cholesterol intake in adults aged >18 years. Horizontal lines represent 95% CIs. Diamonds represent the pooled estimates from the random-effects analysis. ES: effect size, CI: confidence interval; Figure S4: Non-linear analysis of cholesterol intake with risk of pancreatic cancer in adults over 18 years of age among case-control studies; Figure S5: Forest plot for the risk of pancreatic cancer based on 20 g/day increase in total fat intake in adults aged >18 years. Horizontal lines represent 95% CIs. Diamonds represent the pooled estimates from the random-effects analysis. ES: effect size, CI: confidence interval; Figure S6: Non-linear analysis of total fat intake with risk of pancreatic cancer in adults over 18 years of age among cohort studies; Figure S7: Forest plot for the risk of pancreatic cancer based on 20 g/day increase in total fat intake in adults aged >18 years. Horizontal lines represent 95% CIs. Diamonds represent the pooled estimates from the random-effects analysis. ES: effect size, CI: confidence interval; Figure S8: Non-linear analysis of total fat intake with risk of pancreatic cancer in adults over 18 years of age among case-control studies; Figure S9: Random-effects meta-regression plots of the association between cholesterol intake and pancreatic cancer risk in cohort studies. [A] Follow-up duration (years), and [B] baseline body mass index (BMI, kg/m^2^); Figure S10: Random-effects meta-regression plots of the association between total fat intake and pancreatic cancer risk in cohort studies. [A] Follow-up duration (years), and [B] baseline body mass index (BMI, kg/m^2^); Figure S11: Random-effects meta-regression plots of the association between cholesterol intake and pancreatic cancer risk in case-control studies. [A] Follow-up duration (years), and [B] baseline body mass index (BMI, kg/m^2^); Figure S12: Random-effects meta-regression plots of the association between total fat intake and pancreatic cancer risk in case-control studies based on follow-up duration (years). References [6,7,9,12,15,16,17,18,21,32,33,34,35,36,37,38,39,40,41,42,43,44,45,46,47] are cited in the supplementary materials.

## References

[1] Rahib L., Coffin T., Kenner B. (2025). Factors Driving Pancreatic Cancer Survival Rates. Pancreas.

[2] Bray F., Laversanne M., Sung H., Ferlay J., Siegel R.L., Soerjomataram I., Jemal A. (2024). Global cancer statistics 2022: GLOBOCAN estimates of incidence and mortality worldwide for 36 cancers in 185 countries. CA Cancer J. Clin..

[3] Hesami Z., Olfatifar M., Sadeghi A., Zali M.R., Mohammadi-Yeganeh S., Habibi M.A., Ghadir M.R., Houri H. (2024). Global Trend in Pancreatic Cancer Prevalence Rates Through 2040: An Illness-Death Modeling Study. Cancer Med..

[4] Mack S., Koessler T., Bichard P., Frossard J.-L. (2025). Pancreatic Cancer: Epidemiology, Risk Factors, and Prevention. Onco.

[5] Hu J.-X., Zhao C.-F., Chen W.-B., Liu Q.-C., Li Q.-W., Lin Y.-Y., Gao F. (2021). Pancreatic cancer: A review of epidemiology, trend, and risk factors. World J. Gastroenterol..

[6] Ghamarzad Shishavan N., Masoudi S., Mohamadkhani A., Sepanlou S.G., Sharafkhah M., Poustchi H., Mohamadnejad M., Hekmatdoost A., Pourshams A. (2021). Dietary intake of fatty acids and risk of pancreatic cancer: Golestan cohort study. Nutr. J..

[7] Arem H., Mayne S.T., Sampson J., Risch H., Stolzenberg-Solomon R.Z. (2013). Dietary fat intake and risk of pancreatic cancer in the Prostate, Lung, Colorectal and Ovarian Cancer Screening Trial. Ann. Epidemiol..

[8] Ghadirian P., Baillargeon J., Simard A., Perret C. (1995). Food habits and pancreatic cancer: A case-control study of the Francophone community in Montreal, Canada. Cancer Epidemiol. Biomark. Prev..

[9] Heinen M.M., Verhage B.A., Goldbohm R.A., van den Brandt P.A. (2009). Meat and fat intake and pancreatic cancer risk in the Netherlands Cohort Study. Int. J. Cancer.

[10] Philip B., Roland C.L., Daniluk J., Liu Y., Chatterjee D., Gomez S.B., Ji B., Huang H., Wang H., Fleming J.B. (2013). A high-fat diet activates oncogenic Kras and COX2 to induce development of pancreatic ductal adenocarcinoma in mice. Gastroenterology.

[11] Woutersen R.A., Appel M.J., van Garderen-Hoetmer A., Wijnands M.V. (1999). Dietary fat and carcinogenesis. Mutat. Res..

[12] Baghurst P.A., McMichael A.J., Slavotinek A.H., Baghurst K.I., Boyle P., Walker A.M. (1991). A case-control study of diet and cancer of the pancreas. Am. J. Epidemiol..

[13] Wang J., Wang W.J., Zhai L., Zhang D.F. (2015). Association of cholesterol with risk of pancreatic cancer: A meta-analysis. World J. Gastroenterol..

[14] Shen Q.-W., Yao Q.-Y. (2015). Total fat consumption and pancreatic cancer risk: A meta-analysis of epidemiologic studies. Eur. J. Cancer Prev..

[15] Thiébaut A.C., Jiao L., Silverman D.T., Cross A.J., Thompson F.E., Subar A.F., Hollenbeck A.R., Schatzkin A., Stolzenberg-Solomon R.Z. (2009). Dietary fatty acids and pancreatic cancer in the NIH-AARP diet and health study. J. Natl. Cancer Inst..

[16] Lin Y., Tamakoshi A., Hayakawa T., Naruse S., Kitagawa M., Ohno Y. (2005). Nutritional factors and risk of pancreatic cancer: A population-based case-control study based on direct interview in Japan. J. Gastroenterol..

[17] Jansen R.J., Robinson D.P., Frank R.D., Anderson K.E., Bamlet W.R., Oberg A.L., Rabe K.G., Olson J.E., Sinha R., Petersen G.M. (2014). Fatty acids found in dairy, protein and unsaturated fatty acids are associated with risk of pancreatic cancer in a case-control study. Int. J. Cancer.

[18] Li D., Zheng J., Hatia R., Hassan M., Daniel C.R. (2022). Dietary Intake of Fatty Acids and Risk of Pancreatic Cancer: A Case-Control Study. J. Nutr..

[19] Luu H.N., Paragomi P., Jin A., Wang R., Neelakantan N., van Dam R.M., Brand R.E., Koh W.P., Yuan J.M. (2021). Quality Diet Index and Risk of Pancreatic Cancer: Findings from the Singapore Chinese Health Study. Cancer Epidemiol. Biomark. Prev..

[20] Petrick J.L., Castro-Webb N., Gerlovin H., Bethea T.N., Li S., Ruiz-Narváez E.A., Rosenberg L., Palmer J.R. (2020). A Prospective Analysis of Intake of Red and Processed Meat in Relation to Pancreatic Cancer among African American Women. Cancer Epidemiol. Biomark. Prev..

[21] Tayyem R.F., Ibrahim M.O., Abuhijleh H., Alatrash R.M., Al-Jaberi T., Hushki A., Albtoush Y., Yacoub S., Allehdan S. (2022). Macronutrients Not Micronutrients Are Associated with the Risk of Pancreatic Cancer: A Jordanian Case-Control Study. Pancreas.

[22] Moher D., Liberati A., Tetzlaff J., Altman D.G. (2009). Preferred reporting items for systematic reviews and meta-analyses: The PRISMA statement. BMJ.

[23] Wells G.A., Shea B., O’Connell D., Peterson J., Welch V., Losos M., Tugwell P. (2011). The Newcastle-Ottawa Scale (NOS) for Assessing the Quality of Nonrandomised Studies in Meta-Analyses [Internet]. https://ohri.ca/en/who-we-are/core-facilities-and-platforms/ottawa-methods-centre/newcastle-ottawa-scale.

[24] DerSimonian R., Laird N. (2015). Meta-analysis in clinical trials revisited. Contemp. Clin. Trials.

[25] Huedo-Medina T.B., Sánchez-Meca J., Marín-Martínez F., Botella J. (2006). Assessing heterogeneity in meta-analysis: Q statistic or I2 index?. Psychol. Methods.

[26] Greenland S., Longnecker M.P. (1992). Methods for trend estimation from summarized dose-response data, with applications to meta-analysis. Am. J. Epidemiol..

[27] Harrell F.E. (2001). Regression Modeling Strategies: With Applications to Linear Models, Logistic Regression, and Survival Analysis.

[28] Crippa A., Discacciati A., Bottai M., Spiegelman D., Orsini N. (2019). One-stage dose-response meta-analysis for aggregated data. Stat. Methods Med. Res..

[29] Orsini N., Li R., Wolk A., Khudyakov P., Spiegelman D. (2012). Meta-analysis for linear and nonlinear dose-response relations: Examples, an evaluation of approximations, and software. Am. J. Epidemiol..

[30] Egger M., Smith G.D., Schneider M., Minder C. (1997). Bias in meta-analysis detected by a simple, graphical test. BMJ.

[31] Begg C.B., Mazumdar M. (1994). Operating characteristics of a rank correlation test for publication bias. Biometrics.

[32] Michaud D.S., Giovannucci E., Willett W.C., Colditz G.A., Fuchs C.S. (2003). Dietary meat, dairy products, fat, and cholesterol and pancreatic cancer risk in a prospective study. Am. J. Epidemiol..

[33] Nöthlings U., Wilkens L.R., Murphy S.P., Hankin J.H., Henderson B.E., Kolonel L.N. (2005). Meat and fat intake as risk factors for pancreatic cancer: The multiethnic cohort study. J. Natl. Cancer Inst..

[34] Meinhold C.L., Dodd K.W., Jiao L., Flood A., Shikany J.M., Genkinger J.M., Hayes R.B., Stolzenberg-Solomon R.Z. (2010). Available carbohydrates, glycemic load, and pancreatic cancer: Is there a link?. Am. J. Epidemiol..

[35] Chan J.M., Wang F., Holly E.A. (2007). Pancreatic cancer, animal protein and dietary fat in a population-based study, San Francisco Bay Area, California. Cancer Causes Control.

[36] Ghadirian P., Simard A., Baillargeon J., Maisonneuve P., Boyle P. (1991). Nutritional factors and pancreatic cancer in the francophone community in Montréal, Canada. Int. J. Cancer.

[37] Howe G.R., Jain M., Miller A.B. (1990). Dietary factors and risk of pancreatic cancer: Results of a Canadian population-based case-control study. Int. J. Cancer.

[38] Olsen G.W., Mandel J.S., Gibson R.W., Wattenberg L.W., Schuman L.M. (1991). Nutrients and pancreatic cancer: A population-based case-control study. Cancer Causes Control.

[39] Farrow D.C., Davis S. (1990). Diet and the risk of pancreatic cancer in men. Am. J. Epidemiol..

[40] Zhang J., Dhakal I.B., Gross M.D., Lang N.P., Kadlubar F.F., Harnack L.J., Anderson K.E. (2009). Physical activity, diet, and pancreatic cancer: A population-based, case-control study in Minnesota. Nutr. Cancer.

[41] Hu J., La Vecchia C., de Groh M., Negri E., Morrison H., Mery L. (2012). Dietary cholesterol intake and cancer. Ann. Oncol..

[42] Stolzenberg-Solomon R.Z., Pietinen P., Taylor P.R., Virtamo J., Albanes D. (2002). Prospective study of diet and pancreatic cancer in male smokers. Am. J. Epidemiol..

[43] Kalapothaki V., Tzonou A., Hsieh C.C., Karakatsani A., Trichopoulou A., Toupadaki N., Trichopoulos D. (1993). Nutrient intake and cancer of the pancreas: A case-control study in Athens, Greece. Cancer Causes Control.

[44] Lucenteforte E., Talamini R., Bosetti C., Polesel J., Franceschi S., Serraino D., Negri E., La Vecchia C. (2010). Macronutrients, fatty acids, cholesterol and pancreatic cancer. Eur. J. Cancer.

[45] Zatonski W., Przewozniak K., Howe G.R., Maisonneuve P., Walker A.M., Boyle P. (1991). Nutritional factors and pancreatic cancer: A case-control study from south-west Poland. Int. J. Cancer.

[46] Bueno de Mesquita H.B., Maisonneuve P., Runia S., Moerman C.J. (1991). Intake of foods and nutrients and cancer of the exocrine pancreas: A population-based case-control study in The Netherlands. Int. J. Cancer.

[47] Bueno de Mesquita H.B., Moerman C.J., Runia S., Maisonneuve P. (1990). Are energy and energy-providing nutrients related to exocrine carcinoma of the pancreas?. Int. J. Cancer.

[48] Feng H.Y., Chen Y.C. (2016). Role of bile acids in carcinogenesis of pancreatic cancer: An old topic with new perspective. World J. Gastroenterol..

[49] King R.J., Singh P.K., Mehla K. (2022). The cholesterol pathway: Impact on immunity and cancer. Trends Immunol..

[50] Sedgwick P. (2015). Bias in observational study designs: Case-control studies. BMJ.

[51] Al Masri M.K., Hasan M.K., Ali S.A., Alsubaiei A.A., Ali S.A., AlMaazmi A.M., Saleh S.A.K., Adly H.M., Kutbi E., AlJurayyan A.N. (2026). Dietary fatty acid intake and the risk of pancreatic cancer: A dose-response meta-analysis of observational studies. Ann. Med. Surg..

[52] Nettleton J.A., Villalpando S., Cassani R.S., Elmadfa I. (2013). Health significance of fat quality in the diet. Ann. Nutr. Metab..

[53] Trajkovic-Arsic M., Kalideris E., Siveke J.T. (2013). The role of insulin and IGF system in pancreatic cancer. J. Mol. Endocrinol..

[54] Zhong W., Wang X., Wang Y., Sun G., Zhang J., Li Z. (2023). Obesity and endocrine-related cancer: The important role of IGF-1. Front. Endocrinol..

[55] Dey T., Mukherjee A., Chakraborty S. (2020). A Practical Overview of Case-Control Studies in Clinical Practice. Chest.

