# Supplementary material for: Association Between Dietary Total Fat and Cholesterol Intake and Pancreatic Cancer Risk: A Dose–Response Meta-Analysis of Cohort and Case-Control Studies
Source: Nutrients. 2026 Aug 16;18(16):2674. doi: 10.3390/nu18162674 (PMC13516841; doi:10.3390/nu18162674)

Supplemental Figure S1. Forest plot for the risk of pancreatic cancer based on 100 mg/day increase in cholesterol intake in adults aged >18 years. Horizontal lines represent 95% CIs. Diamonds represent the pooled estimates from the random-effects analysis. ES: effect size, CI: confidence interval.

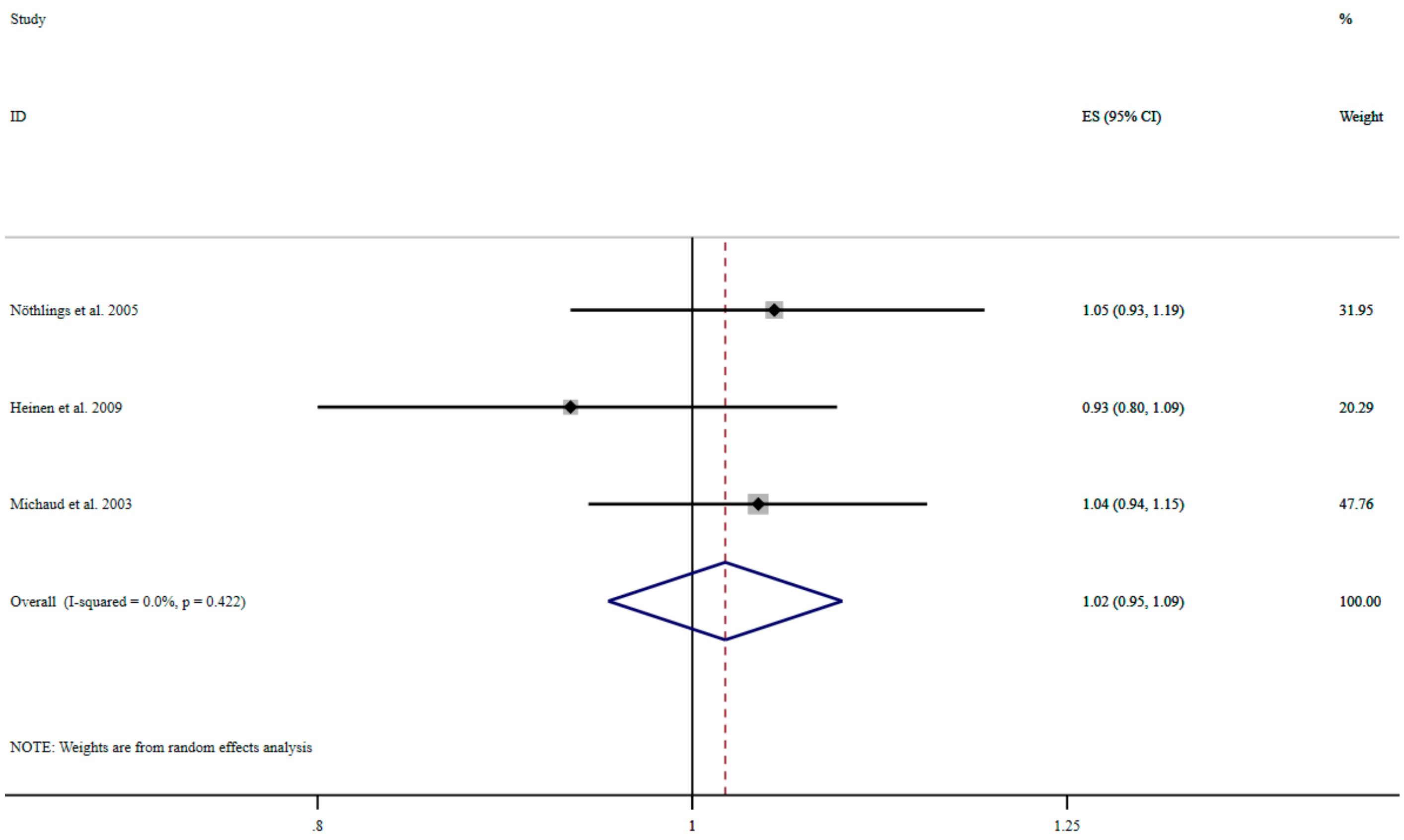

Supplemental Figure S2. Non-linear analysis of cholesterol intake with risk of pancreatic cancer in adults over 18 years of age among cohort studies.

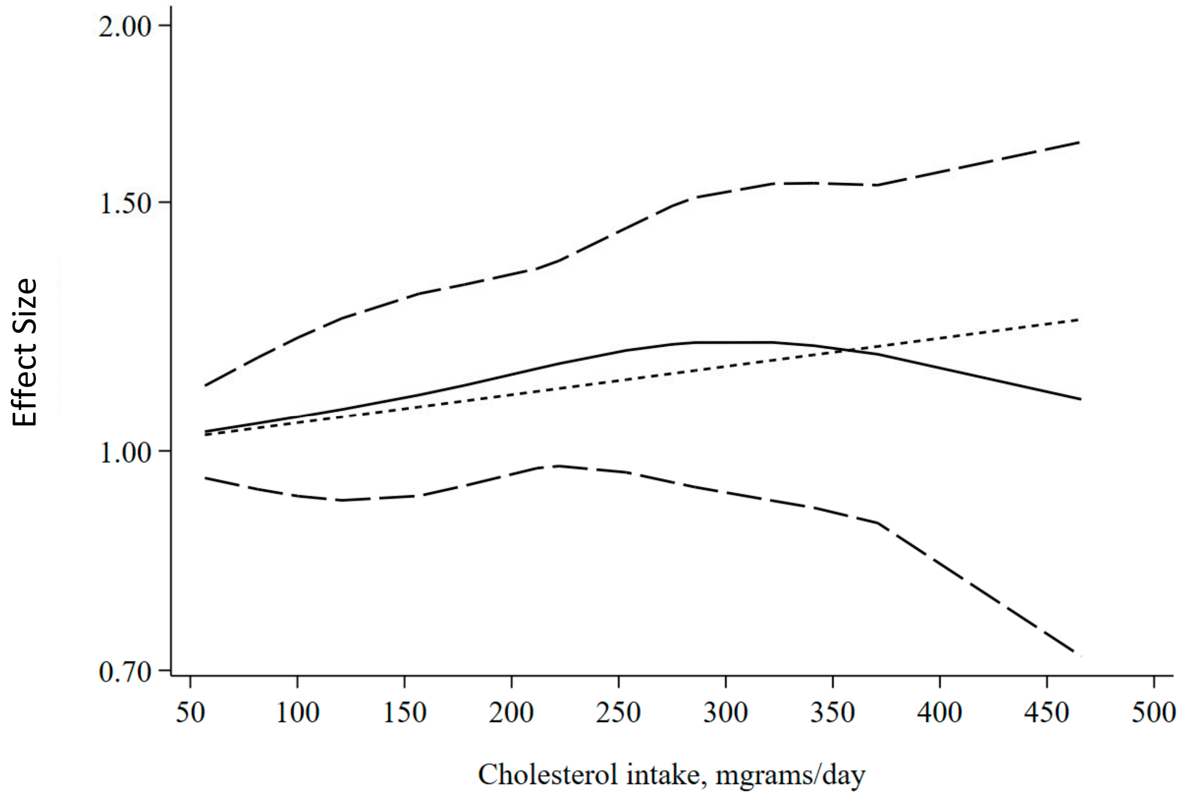

Supplemental Figure S3. Forest plot for the risk of pancreatic cancer based on 100 mg/day increase in cholesterol intake in adults aged >18 years. Horizontal lines represent 95% CIs. Diamonds represent the pooled estimates from the random-effects analysis. ES: effect size, CI: confidence interval.

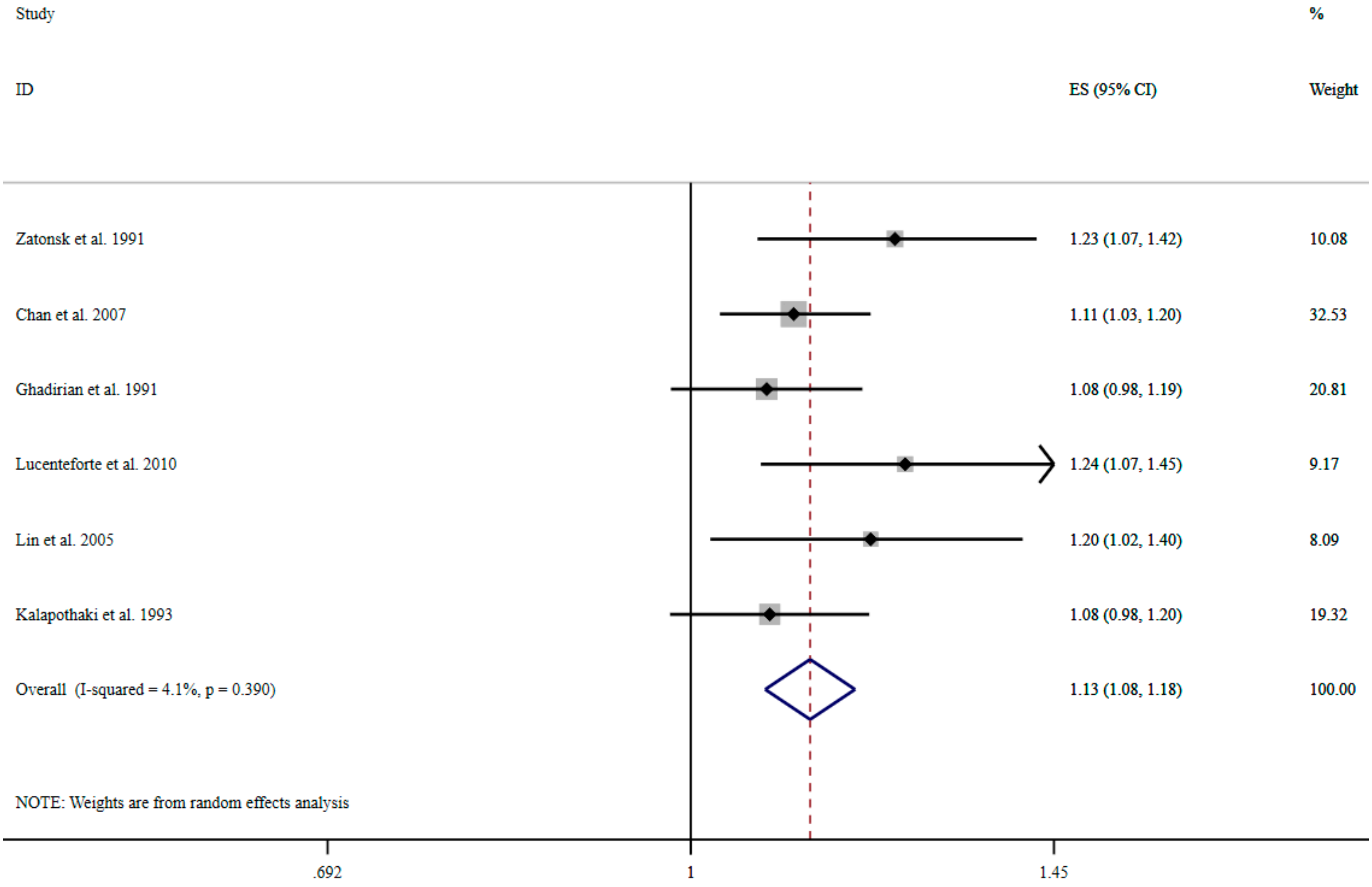

Supplemental Figure S4. Non-linear analysis of cholesterol intake with risk of pancreatic cancer in adults over 18 years of age among case-control studies.

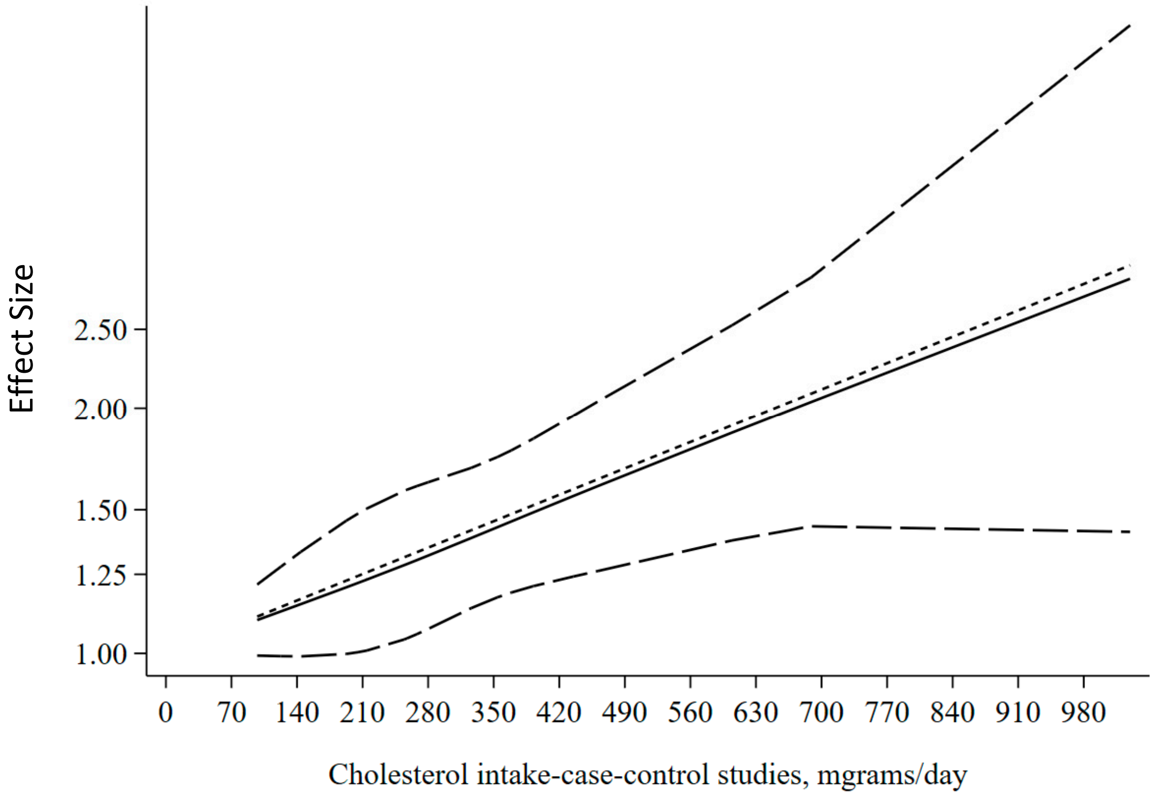

Supplemental Figure S5. Forest plot for the risk of pancreatic cancer based on 20 g/day increase in total fat intake in adults aged >18 years. Horizontal lines represent 95% CIs. Diamonds represent the pooled estimates from the random-effects analysis. ES: effect size, CI: confidence interval.

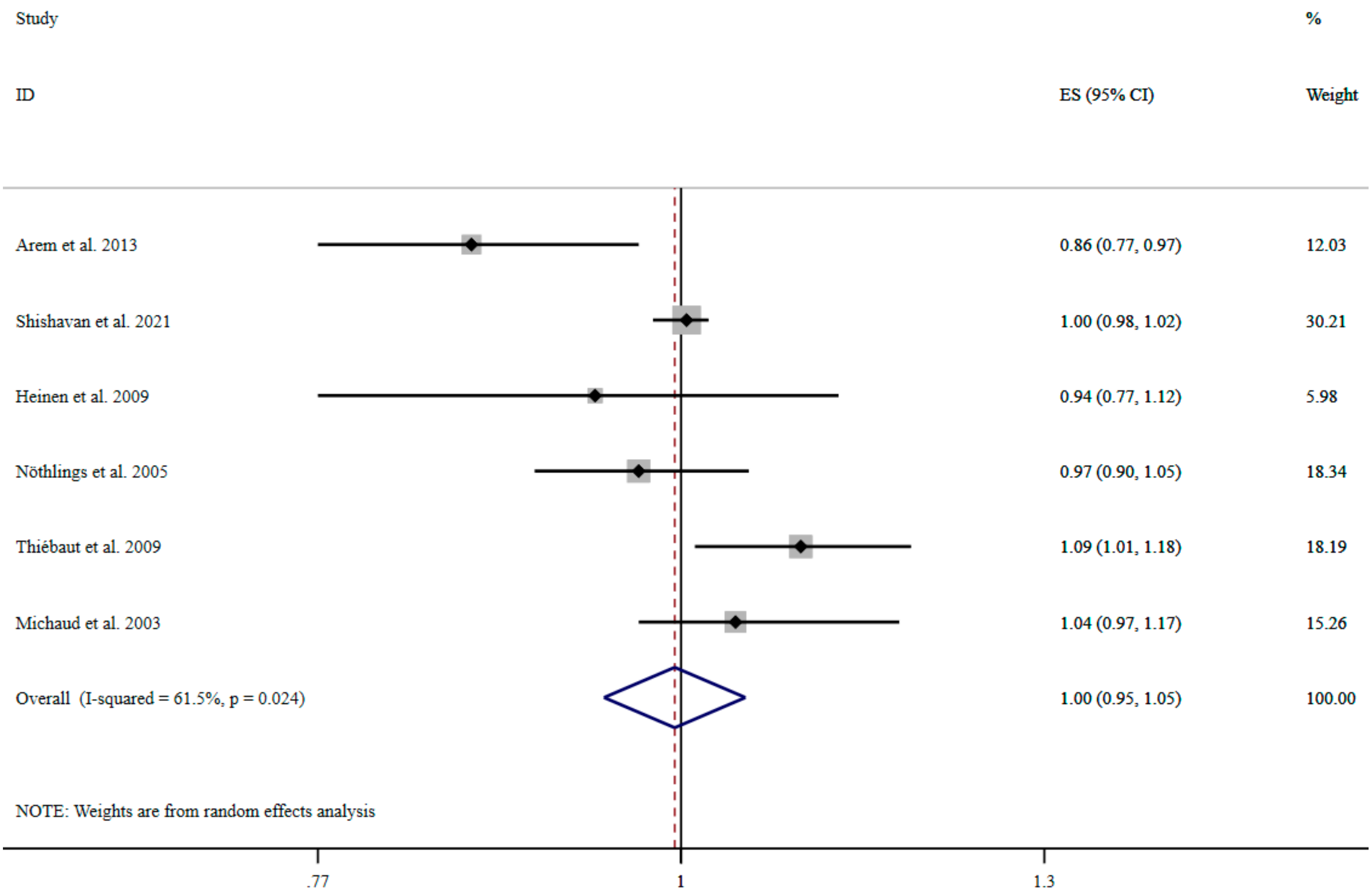

Supplemental Figure S6. Non-linear analysis of total fat intake with risk of pancreatic cancer in adults over 18 years of age among cohort studies.

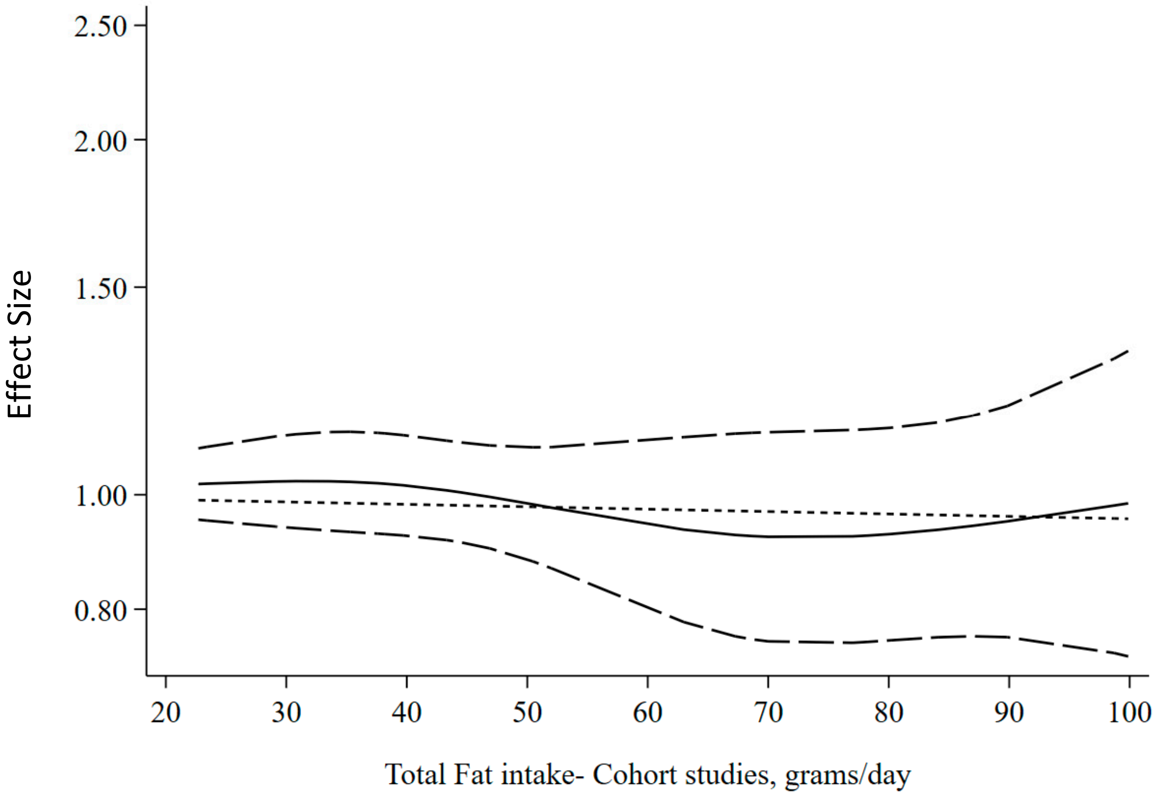

Supplemental Figure S7. Forest plot for the risk of pancreatic cancer based on 20 g/day increase in total fat intake in adults aged >18 years. Horizontal lines represent 95% CIs. Diamonds represent the pooled estimates from the random-effects analysis. ES: effect size, CI: confidence interval.

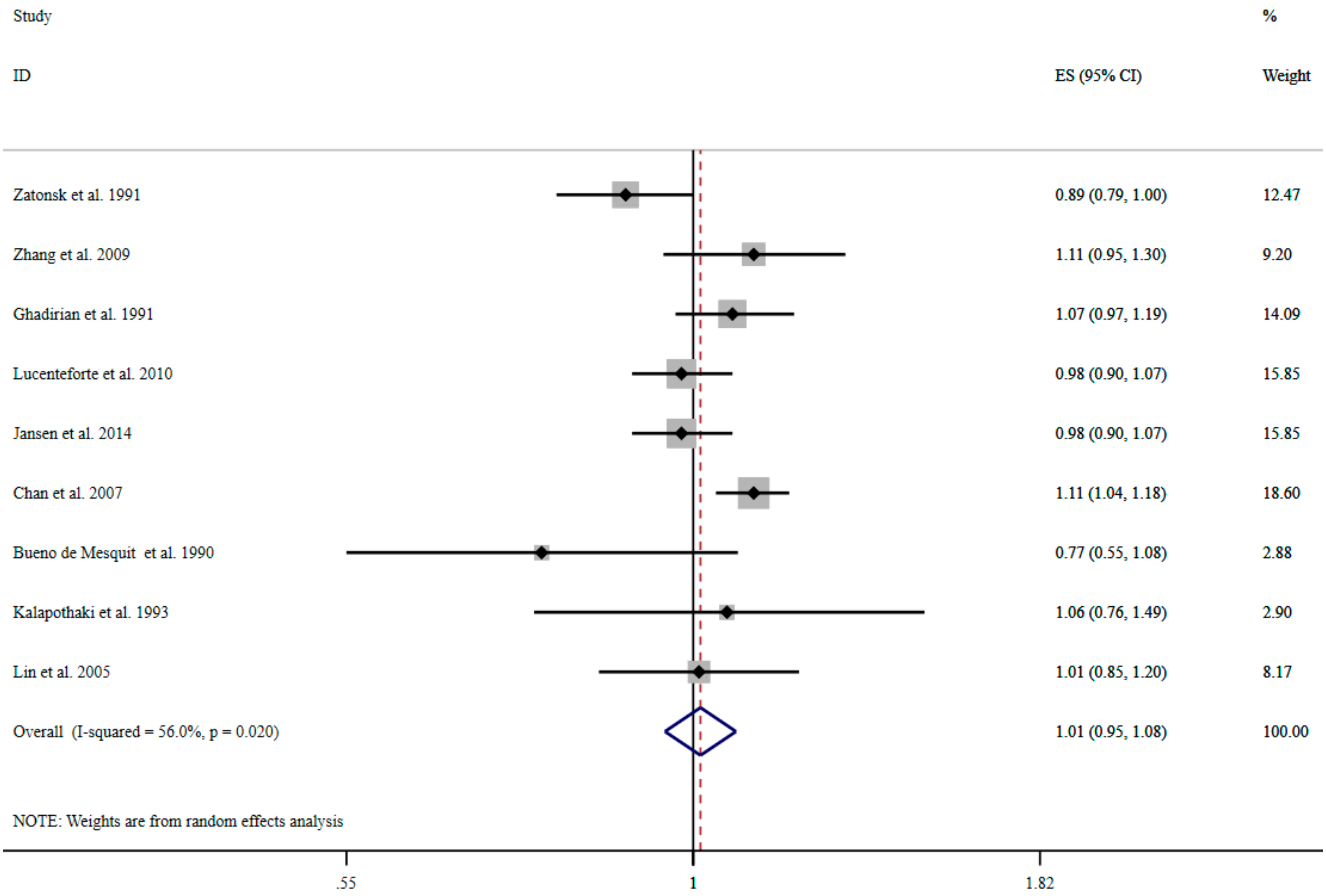

Supplemental Figure S8. Non-linear analysis of total fat intake with risk of pancreatic cancer in adults over 18 years of age among case-control studies.

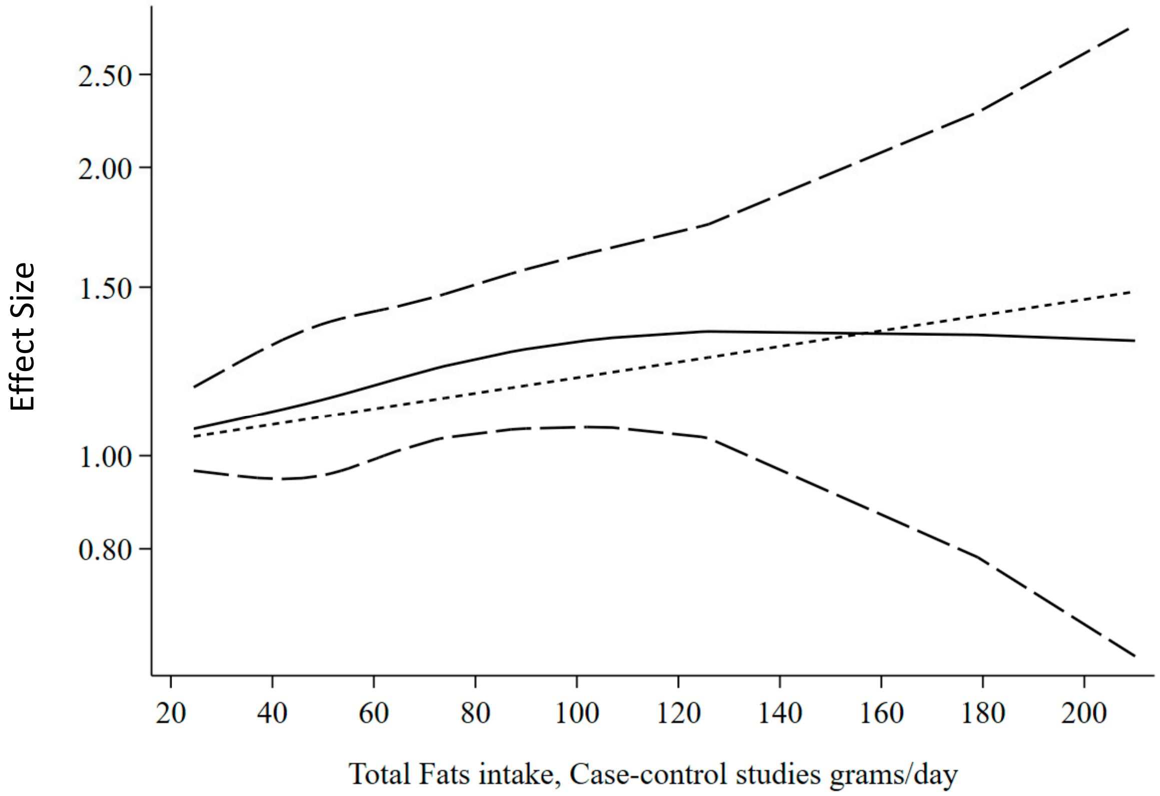

Supplemental Figure S9. Random-effects meta-regression plots of the association between cholesterol intake and pancreatic cancer risk in cohort studies. [A] Follow-up duration (years), and [B] baseline body mass index (BMI, kg/m²).

[A]

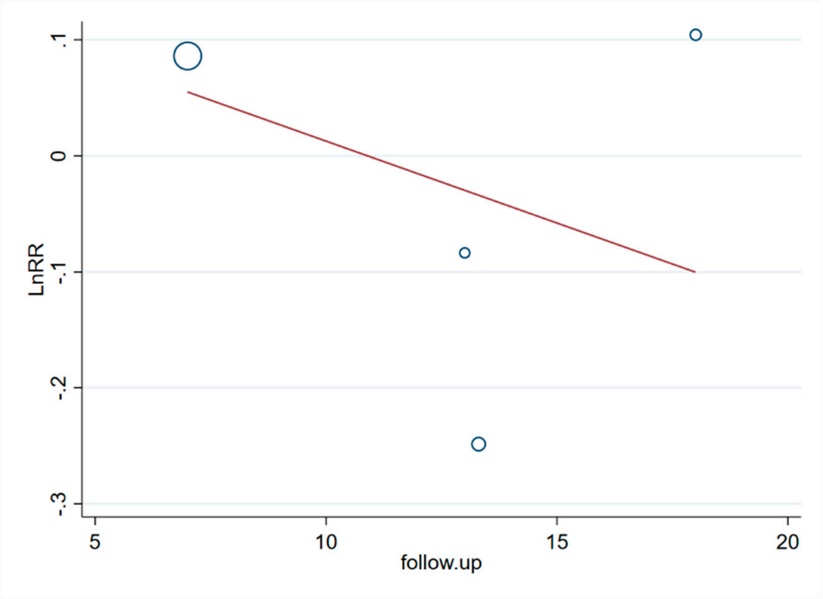

[B]

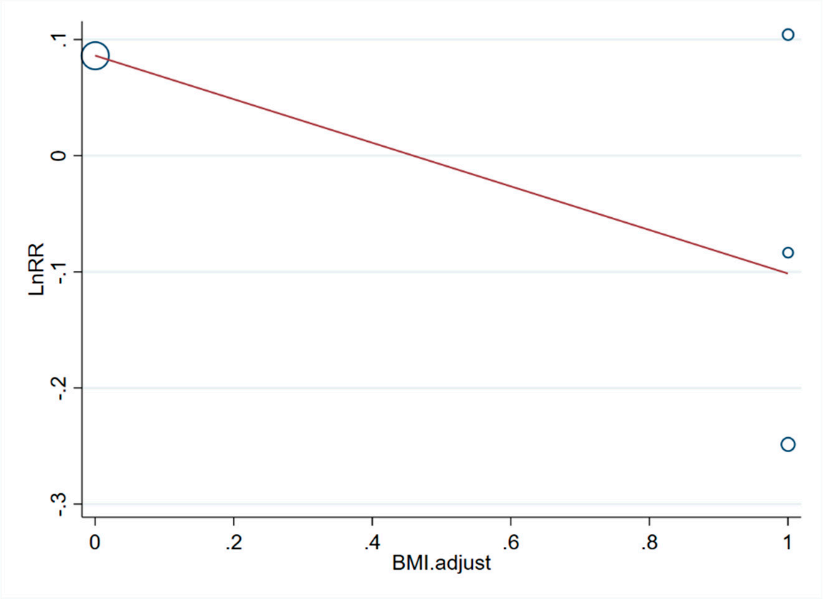

Supplemental Figure S10. Random-effects meta-regression plots of the association between total fat intake and pancreatic cancer risk in cohort studies. [A] Follow-up duration (years), and [B] baseline body mass index (BMI, kg/m²).

[A]

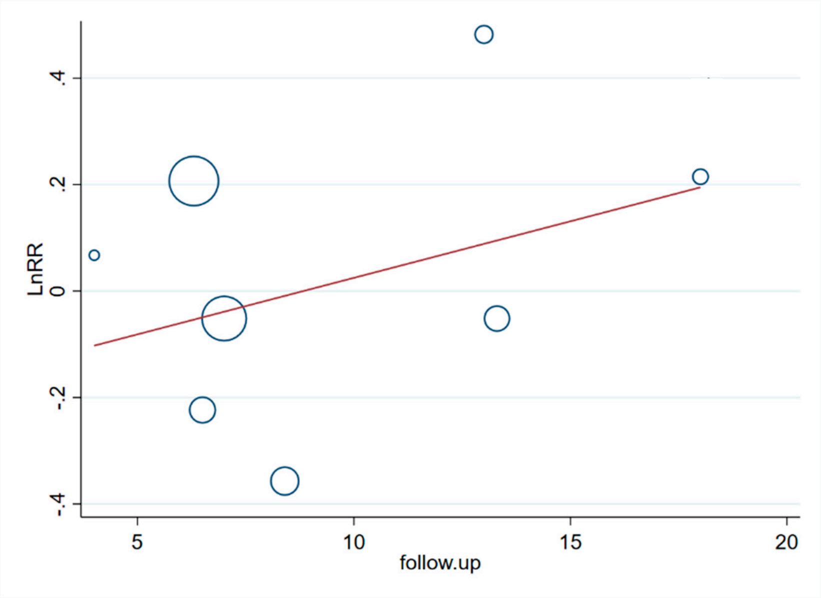

[B]

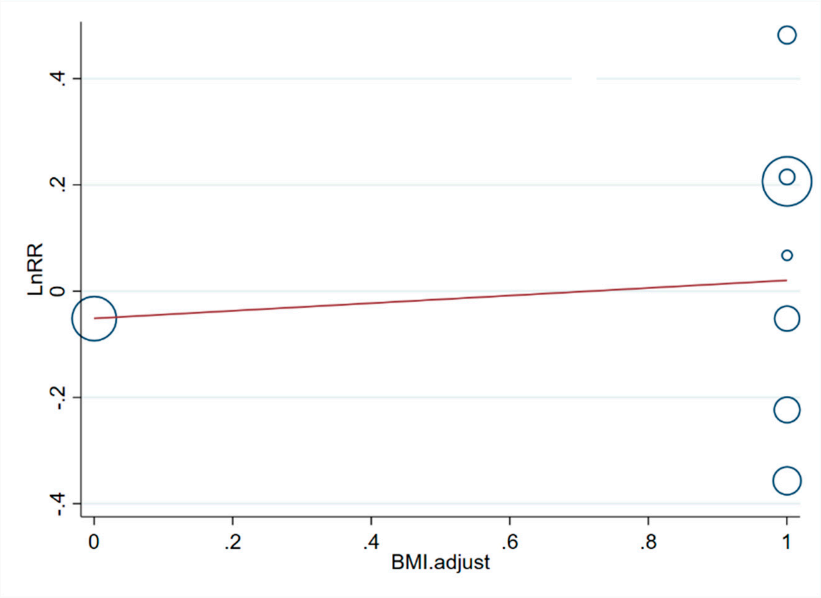

Supplemental Figure S11. Random-effects meta-regression plots of the association between cholesterol intake and pancreatic cancer risk in case-control studies. [A] Follow-up duration (years), and [B] baseline body mass index (BMI, kg/m²).

[A]

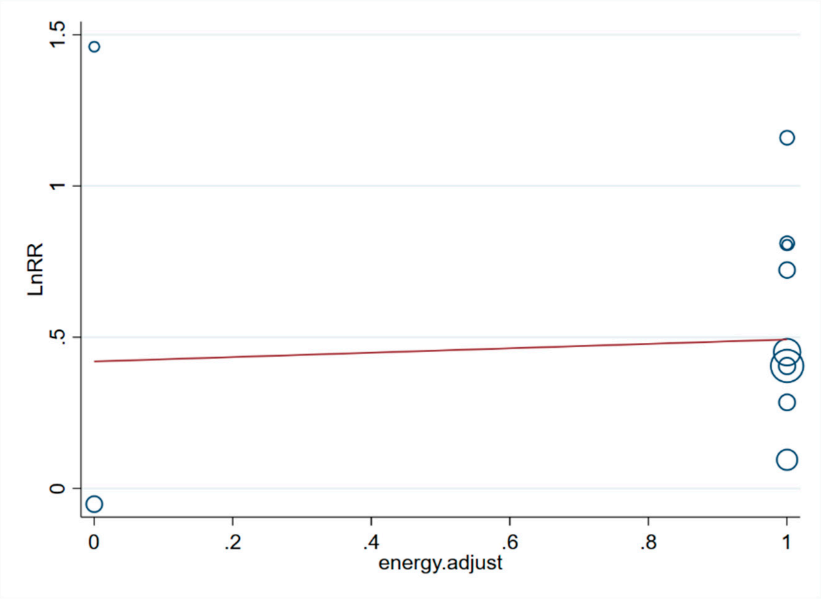

[B]

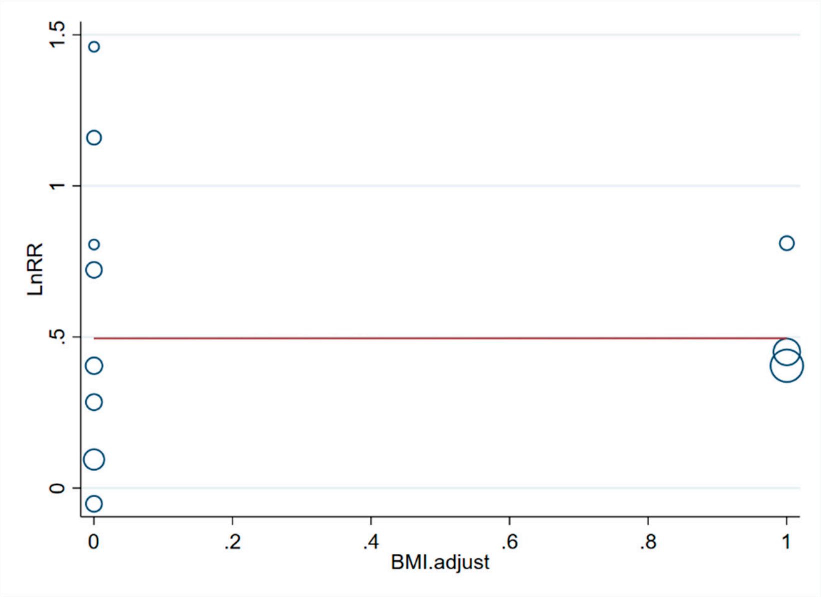

Supplemental Figure S12. **R**andom-effects meta-regression plots of the association between total fat intake and pancreatic cancer risk in case-control studies based on follow-up duration (years).

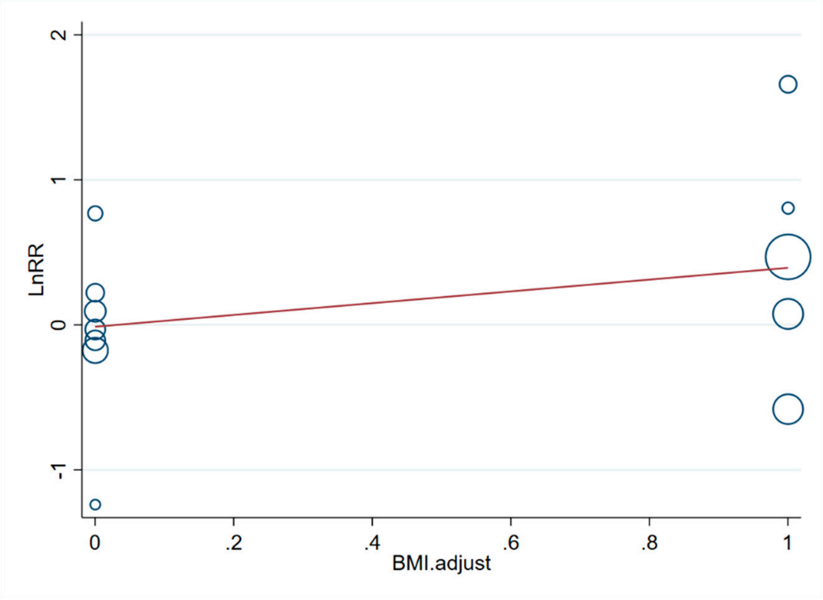

Supplement: Supplementary file 1 [file nutrients-18-02674-s001.zip › PC_Supplementary Figures_08-03-26.pdf]
