# Supplementary material for: Association Between Dietary Total Fat and Cholesterol Intake and Pancreatic Cancer Risk: A Dose–Response Meta-Analysis of Cohort and Case-Control Studies
Source: Nutrients. 2026 Aug 16;18(16):2674. doi: 10.3390/nu18162674 (PMC13516841; doi:10.3390/nu18162674)
Supplement: Supplementary file 1 [file nutrients-18-02674-s001.zip › PC_Supplementary Tables_08-03-26.pdf]

Supplementary Table S1. Search strategy for searching articles in scientific databases.

Database	Search query	Citation number
PubMed	"Dietary Fat"[Title/Abstract] OR "Unsaturated Dietary Fat"[Title/Abstract] OR "Dietary Oils"[Title/Abstract] OR "Dietary Oil"[Title/Abstract] OR "fat"[Title/Abstract] OR "fatty"[Title/Abstract] OR "monounsaturated fatty acid"[Title/Abstract] OR "polyunsaturated fatty acid"[Title/Abstract] OR "MUFA"[Title/Abstract] OR "PUFA"[Title/Abstract] OR "saturated fatty acid"[Title/Abstract] OR "SFA"[Title/Abstract] OR "trans fatty acid"[Title/Abstract] OR "fat intake"[Title/Abstract] OR "high-fat diet"[Title/Abstract] OR "linoleic"[Title/Abstract] OR "linolenic"[Title/Abstract] OR "eicosapentaenoic"[Title/Abstract] OR "arachidonic"[Title/Abstract] OR "EPA"[Title/Abstract] OR "docosahexaenoic"[Title/Abstract] OR "DHA"[Title/Abstract] OR "trans"[Title/Abstract] OR "fatty acids"[Title/Abstract] OR "elaidic"[Title/Abstract] OR "cholesterol"[Title/Abstract] OR "lipids"[Title/Abstract] OR "total cholesterol"[Title/Abstract] OR "dietary cholesterol"[Title/Abstract] OR "cholesterol intake"[Title/Abstract] OR "Dietary Fat"[Title/Abstract] OR "fat intake"[Title/Abstract]	1,286,888
	"Pancreatic Neoplasm"[Title/Abstract] OR "Pancreas Neoplasms"[Title/Abstract] OR "Pancreas Neoplasm"[Title/Abstract] OR "Cancer of Pancreas"[Title/Abstract] OR "Pancreas Cancer"[Title/Abstract] OR "Pancreatic Cancer"[Title/Abstract] OR "Cancer of the Pancreas"[Title/Abstract]	67,777
	"case control"[Title/Abstract] OR "cohort"[Title/Abstract] OR "prospective"[Title/Abstract] OR "retrospective"[Title/Abstract] OR "epidemiology"[Title/Abstract]	2,806,945
	#1 AND #2 AND #3	350
Scopus	TITLE-ABS-KEY ("Dietary Fat" OR "Unsaturated Dietary Fat" OR "Dietary Oils" OR "Dietary Oil" OR "fat" OR "fatty" OR "monounsaturated fatty acid" OR "polyunsaturated fatty acid" OR "MUFA" OR "PUFA" OR "saturated fatty acid" OR "SFA" OR "trans fatty acid" OR "fat intake" OR "high-fat diet" OR "linoleic" OR "linolenic" OR "eicosapentaenoic" OR "arachidonic" OR "EPA" OR "docosahexaenoic" OR "DHA" OR "trans" OR "fatty acids" OR "elaidic" OR "cholesterol" OR "lipids" OR "total cholesterol" OR "dietary cholesterol" OR "cholesterol intake" OR "Dietary Fat" OR "fat intake")	3,231,100
	TITLE-ABS-KEY ("Pancreatic Neoplasm" OR "Pancreas Neoplasms" OR "Pancreas Neoplasm" OR "Cancer of Pancreas" OR "Pancreas Cancer" OR "Pancreatic Cancer" OR "Cancer of the Pancreas")	158,230
	TITLE-ABS-KEY ("case control" OR "cohort" OR "prospective" OR "retrospective" OR "epidemiology")	5,180,075
	#1 AND #2 AND #3	1,644
Web of Science	"Dietary Fat" OR "Unsaturated Dietary Fat" OR "Dietary Oils" OR "Dietary Oil" OR "fat" OR "fatty" OR "monounsaturated fatty acid" OR "polyunsaturated fatty acid" OR "MUFA" OR "PUFA" OR "saturated fatty acid" OR "SFA" OR "trans fatty acid" OR "fat intake" OR "high-fat diet" OR "linoleic" OR "linolenic" OR "eicosapentaenoic" OR "arachidonic" OR "EPA" OR "docosahexaenoic" OR "DHA" OR "trans" OR "fatty acids" OR "elaidic" OR "cholesterol" OR "lipids" OR "total cholesterol" OR "dietary cholesterol" OR "cholesterol intake" OR "Dietary Fat" OR "fat intake" (Topic)	1,345,595
	"Pancreatic Neoplasm" OR "Pancreas Neoplasms" OR "Pancreas Neoplasm" OR "Cancer of Pancreas" OR "Pancreas Cancer" OR "Pancreatic Cancer" OR "Cancer of the Pancreas" (Topic)	87,866
	"case control" OR "cohort" OR "prospective" OR "retrospective" OR "epidemiology" (Topic)	2,761,851
	#1 AND #2 AND #3	503
Google Scholar	"Dietary Fat" AND "Pancreas Cancer"	100

Supplementary Table S2. The quality of cohort studies using the Newcastle-Ottawa Scale.

Author	Representativeness of the exposed cohort	Selection of the non-exposed cohort	Ascertainment of exposure	Outcome of interest was not present at start of study	Energy adjustment	Controls for any additional factor	Assessment of outcome	Follow-up long enough	Adequacy of follow up of cohorts	Total
Heinen et al. 2009	*	*	*	*	*	*	*	*	*	9
Michaud et al. 2003	*	*	*	*	*	*	*	*	*	9
Nöthlings et al. 2005	*	*	*	*	*		*	*	*	8
Stolzenberg-Solomon et al. 2002	*	*	*	*	*	*	*	*	*	9
Shishavan et al. 2021	*	*		*	*	*	*		*	7
Arem et al. 2013	*	*	*	*	*	*	*	*	*	9
Thiébaut et al. 2009	*	*	*	*	*	*	*		*	8
Meinhold et al. 2009	*	*	*	*	*	*	*		*	8

Supplementary Table S3. The quality of case-control studies using the Newcastle-Ottawa Scale.

Author	Case definition adequate	Representativeness of the cases	Selection of Controls	Definition of Controls	Adjustment for energy	Controls for any addition al factor	Ascertainment of exposure	Ascertainment for cases and controls	Non-Response rate	Total
Baghurst et al. 1991	*	*	*	*	*	*		*		7
Chan et al. 2007	*	*	*	*	*	*	*	*	*	9
Lin et al. 2005	*	*	*	*	*		*	*		7
Ghadirian et al. 1991	*	*	*	*	*			*		6
Howe et al. 1990	*	*	*	*	*			*		6
Kalapothaki et al. 1993	*		*	*	*	*		*		7
Lucenteforte et al. 2010	*	*	*	*	*		*	*	*	8
Olsen et al. 1991	*	*	*	*	*	*		*		7
Tayyem et al. 2022	*	*	*	*	*	*		*	*	8
Zatonsk et al. 1991	*	*	*	*	*			*		6
de Mesquit et al. 1991	*	*	*	*				*	*	6
Jansen et al. 2014	*	*		*	*	*	*	*		7
Hu et al. 2012	*	*	*	*	*	*		*		7
Bueno de Mesquit et al. 1990(A)	*	*	*	*				*	*	6
Bueno de Mesquit et al.1990(B)	*	*	*	*				*	*	6
Li et al. 2022	*	*	*	*	*		*	*	*	8
Farrow and Davis et al 1990	*	*	*	*	*	*	*	*		8
Zhang et al. 2009	*	*	*	*	*	*		*	*	8
